# Economic evaluation of artificial intelligence for cancer detection in the UK breast screening programme

**DOI:** 10.1038/s41416-026-03465-3

**Published:** 2026-05-02

**Authors:** Harry Hill, Cristina Roadevin

**Affiliations:** 1 School of Medicine and Population Health, University of Sheffield, Sheffield, UK; 2 School of Medicine, University of Nottingham, Nottingham, UK

**Keywords:** Health care economics, Health policy, Population screening

## Abstract

**Background:**

Artificial intelligence (AI) offers a potential solution to radiologist shortages in breast cancer screening while maintaining diagnostic accuracy. Retrospective studies suggest AI performs comparably to human readers in detecting cancers, but no economic evaluations have yet used prospective trial data.

**Methods:**

We developed a de novo discrete-event simulation model to estimate the cost-effectiveness of integrating AI into the NHS screening pathway using evidence from a large prospective trial.

**Results:**

The AI-only strategy generated a small incremental QALY gain of 0.00009 and reduced lifetime costs by £159.55 per woman invited, and had a 100% probability of being most cost-effective at the £20,000/QALY threshold. Replacing one human reader with AI also increased QALYs, by 0.00019, and reduced costs by £31.07. Triple reading (two humans plus AI) produced the largest QALY gain (0.00023) but increased costs by £72.79. All AI-based pathways reduced cancer deaths, shifted cancers from advanced (TNM stage 4) to earlier stages at detection, and increased the proportion of cancers detected by screening.

**Conclusion:**

Using AI in place of human readers is likely to be cost-effective, marginally improving health outcomes while reducing overall costs, with full replacement of both human readers being the most cost-effective screening strategy.

## Introduction

Breast cancer screening offers important benefits but also carries recognised harms, and its effectiveness has long been the subject of debate [1]. Contemporary evidence indicates that early detection contributes to a reduction in breast cancer mortality [2, 3], although both the magnitude of this benefit and the extent of overdiagnosis remain areas of discussion [1, 4]. An international meta-analysis of cohort studies [3] demonstrated that participation in organised mammography screening programmes reduces incidence-based breast cancer mortality by approximately 20-30%. Evidence also suggests that these mortality gains are delivered at an economically acceptable cost. A systematic review [5] of 32 economic evaluations of breast cancer screening and seven evaluations of primary prevention found that all studies predicted gains in life expectancy at costs considered acceptable within their respective healthcare systems, based on evidence from European screening programmes.

The NHS Breast Screening Programme invites women aged 50 up to their 71st birthday for routine mammography every three years [6]. Each mammogram is double-read, meaning that two radiologists independently interpret the images. Since its launch in 1988, the NHS Breast Screening Programme has been associated with improvements in breast cancer outcomes. Contemporary population-based evidence in England shows lower mortality among women who attend screening, with a national case-control analysis reporting a 38% mortality reduction [7] and another study finding a 21% reduction after adjustment for the healthier-volunteer effect [8]. Concerns about overdiagnosis and the balance of benefits and harms also continue to inform debates on how screening programmes should be organised [1, 9].

These debates are set against a backdrop of operational pressures. The NHS faces a shortage of breast radiologists. Recent workforce data from the Royal College of Radiologists report the UK has a 29% shortfall of consultant clinical radiologists, with around one in four breast radiologists expected to retire within the next five years and a projected shortfall of 39% by 2029 if current trends persist. Screening activity remains high, with 2.5 million women invited and 1.95 million mammograms performed in 2023–24 [10]. Yet the number of accredited breast radiologists has not kept pace [11], leaving some services unable to meet their reading workload and reliant on outsourcing or remote cross-site reading to manage workload [12]. In this operational environment, maintaining timely double reading may be difficult.

Artificial intelligence (AI) has emerged as a potential solution to these workforce pressures, with some retrospective studies indicating that AI can interpret mammograms at a level comparable to radiologist performance [13, 14]. Two economic evaluations of AI-supported mammography have been published [15, 16]. These studies use retrospective evidence of AI performance and find that AI could be cost-effective, and in one study, cost-saving [16]. However, retrospective evidence does not capture the complexities of clinical workflows [14]. Accordingly, such evaluations treat improvements in diagnostic performance as directly translatable into proportional shifts in downstream resource use (e.g., recall rates, biopsy frequencies, consensus reading volumes, and radiologist workload). Real-world screening pathways involve workflow constraints, consensus rules, and safety thresholds that can limit these effects [14]. Therefore, health-economic modelling should ideally draw on prospective evidence of AI performance that captures real-world changes in downstream resource use.

To address the limitations of existing evidence and support policy development, we conducted the first economic evaluation of AI integration into breast cancer screening that incorporates prospective clinical accuracy data of AI performance. We used clinical accuracy data from the ScreenTrustCAD trial [17], a large population-based, paired-reader study conducted within the Swedish national screening programme. We adapted these data to the UK context by incorporating NHS screening workflows and resource use to evaluate the cost-effectiveness of using AI either to augment or to replace human readers.

## Methods

We constructed a de novo discrete-event simulation (DES) model to evaluate the cost-effectiveness of integrating AI for cancer detection in the NHS Breast Screening Programme. The model replicates the UK screening pathway, capturing individual screening and treatment trajectories along with the immediate and long-term benefits of earlier cancer detection, such as cancer stage shift, improved survival, reduced recurrence, and lower treatment costs. The evaluation followed National Institute for Health and Care Excellence (NICE) guidance (2025) [18] and the CHEERS-AI checklist (2024) [19], was conducted from the NHS and personal social services perspective, and considered lifetime costs and outcomes. The model was built in R (version 4.2.2). The model code is available upon request to the corresponding author and may be accessed only for academic and reproducibility purposes with approval from the study’s funder. All model assumptions, technical methods, and data sources are detailed in the appendix.

Four screening strategies were compared based on the configurations evaluated in the ScreenTrustCAD trial. Standard double reading by two radiologists, double reading by one radiologist plus AI, single reading by AI alone, and triple reading by two radiologists plus AI. The AI system used was Insight MMG version 1.1.6 (Lunit, South Korea), with technical details available in the original trial publication [17]. The DES model design was chosen for its ability to represent individual histories and timing of key events, capturing the complexity of population-based breast cancer screening pathways.

### Model structure and overview

**Fig. 1:**
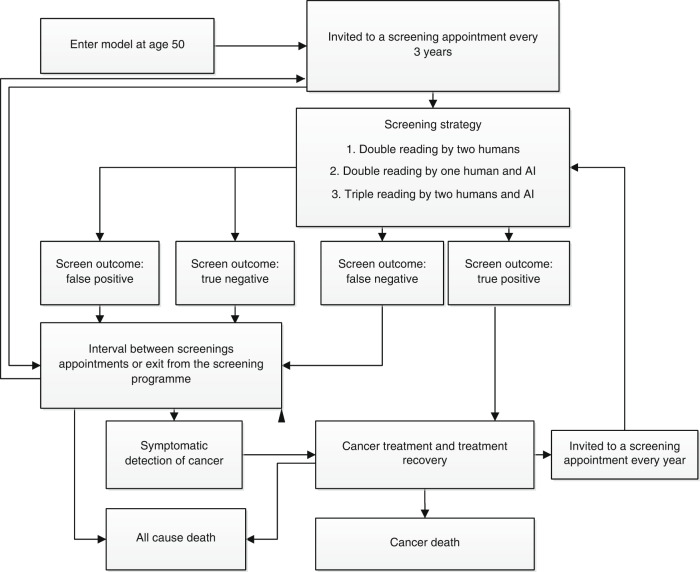
Model structure and screening pathways. The figure shows the structure of the breast screening model, including entry age, screening strategies, screening outcomes, cancer detection, treatment/recovery, cancer death, and all-cause death.

Figure 1 shows the model structure, detailing the sequence of clinical events and potential pathways from the initiation of screening to cancer detection and eventual mortality. The simulated cohort represents women eligible for routine screening. At the model’s start, each individual is assigned an age at death for other causes than breast cancer based on recent national life tables for adult women in England [20]. They are also assigned a cancer status based on the observed 9.05% probability of developing breast cancer between ages 50 and 74 in England [21]. Women are then invited for breast screening at age 50. Those who attend proceed to mammography, which is evaluated using one of the four screening strategies. Cancer detection under each strategy is determined by its respective sensitivity and specificity. The model then stratifies women into different pathways, in line with UK guidelines [6, 22]. Women without a cancer diagnosis are invited for routine screening every three years. Women diagnosed with breast cancer receive the same mammographic imaging modality as in routine screening, but enter a surveillance pathway in which they undergo annual mammography for ten years before returning to three-yearly screening. Screening in both the routine and surveillance pathways ceases once women reach the upper screening age of 71.

**Fig. 2:**
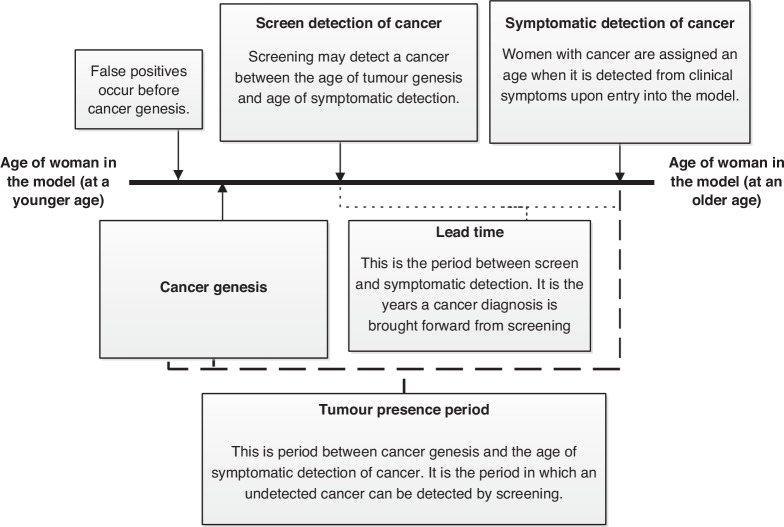
Representation of cancer genesis, detection, and lead time. The figure illustrates the relationship between cancer genesis, screen detection, symptomatic detection, tumour presence period, false positives, and lead time within the model.

We have described how a cancer can be detected at screening appointments. To represent cancers that present between screens or in individuals who do not attend, the model incorporates a natural history sub-model, illustrated in Fig. 2. For women who develop cancer, this includes assigning an age of symptomatic cancer detection. This is drawn from the empirical distribution of the age of screen-detected cancers in the English programme [23], with adjustment to reflect the average delay of 0.8 years between screen-detected and clinically detected presentation observed in recent NHS data [24]. After assigning the symptomatic detection age, the natural history model estimates the underlying tumour onset age by sampling the preclinical detectable phase from distributions reported in contemporary breast cancer natural history modelling and subtracting this duration from the symptomatic age [25]. During this preclinical phase cancers can be detected by screening earlier than they would have appeared symptomatically, creating a lead-time advantage [1] for screen detection. This structure produces individual-level variation in tumour onset and symptomatic presentation, which are specified independently of the screening strategy applied. After diagnosis, whether identified through screening or symptomatically, women transition into treatment pathways and the model allows for the possibility of recurrence for up to 25 years after diagnosis. Each woman is followed until death from breast cancer or other causes.

### Model data sources and model assumptions

The model adopts a single-cohort design, with all women entering the simulation at a common starting age of 50. Screening attendance is modelled probabilistically based on age, invitation type (first or repeat), and screening history, using data from the NHS Breast Screening Programme Audit (2022–2023) [23] and the Age Trial [26]. UK baseline accuracy data for the standard screening strategy vary by age and breast density [27]. Breast density is assessed using the Volpara Density Grade [28] (VDG), an automated volumetric metric based on mammographic x-ray attenuation that assigns breasts to four ordered strata from almost entirely fatty to extremely dense. Diagnostic accuracy for the three AI-based strategies is derived from the Swedish ScreenTrustCAD trial [17]. We applied the relative accuracy changes in the ScreenTrustCAD trial to the matched age–density strata in the UK baseline data [27].

At cancer detection, tumour stage is assigned probabilistically as DCIS or invasive stage 1–4 using age- and detection-mode–specific distributions from UK audit data [29]. In this approach, the stage is determined indirectly through the natural history process. Cancers detected at routine screening follow the stage distribution observed for screen-detected cases, whereas cancers missed at screening, whether they later appear as interval cancers or present symptomatically, are assigned a more advanced stage distribution consistent with older age at diagnosis and the patterns seen in clinically detected disease. Improved mammography and reader performance reduce the number of missed cancers and shift detection to earlier ages and to screen-detected presentations. This indirect approach to stage assignment follows the methodology used in recent discrete-event simulation evaluations of changes to the UK Breast Screening Programme [30, 31].

After stage assignment, ten-year survival is sampled from probabilities by age, stage, and mode of detection drawn from the English breast cancer registry data [2]. Recurrence is sampled from probabilities that vary by stage and time since diagnosis, based on cohort studies of women in England with breast cancer [32, 33]. Annual recurrence is tracked up to 25 years for invasive cancer [32] and 20 years for DCIS [33]. The model assumes recurrent cancers do not appear at a lower stage than the original diagnosis, consistent with published studies [34–36].

The model was run probabilistically, with probability distributions assigned to all input parameters, including diagnostic accuracy, survival, health utility and costs (appendix, pp 51-53).

### Costs and resource use

Costs were estimated in 2023 British Pounds from a UK payer perspective, discounted at 3.5% per NICE guidance [18]. Screening-related costs were costed using NHS reference costs [37]. The mammography imaging cost (£41 per screen in standard screening) reflects the technical cost of the mammogram, including equipment, technologist time, and the single embedded read. The tariff cost does not, however, cover the cost of screening invitations (£0.73 per invite), and the full staffing cost of image interpretation, which in standard practice involves two independent reads and, where these disagree, an additional consensus read. Diagnostic follow-up procedures include ultrasound (£68), biopsy (£373–£400), and MRI (£392). These tariffs include staff time for both the procedure and its interpretation, and therefore, their cost does not vary across screening strategies. AI-specific costs include a per-screen licensing fee (£2.02, as recommended by NICE [38]), as well as costs for IT infrastructure, governance, and staff time associated with implementation and oversight [39] (£3.89 per screen, based on NHS pilot data). Screen reader staffing costs for mammography were calculated using micro-costing methods based on national average salaries for radiologists (£113,962) and radiographers (£45,600), adjusted for role, region, experience, and locum use [40]. These were further weighted for actual staff mix, workload, and arbitration requirements, resulting in a per-read reader staffing cost of £25.11. Staffing costs varied by intervention. The single reader plus AI pathway uses one reader per screen, with a second reader only for AI disagreement. In contrast, the per-read costs are doubled in the standard screening pathway because every case is double-read and a third reader is used for disagreement. Cancer treatment costs were stratified by cancer stage and time since diagnosis using UK patient-level costing data [41, 42]. End-of-life care costs were determined by cause and age of death [43].

### Health-related quality of life and clinical outcomes

Health outcomes are expressed as quality-adjusted life-years (QALYs) derived from the EQ-5D instrument, following NICE 2025 guidance [18], and discounted at 3.5% per year. Additionally, clinical outcomes measured include tumour stage at detection, cancer deaths, and the proportion of cancers detected through screening. To accurately capture health-related quality of life, baseline utilities for women of screening age are drawn from UK population norms [44, 45]. Utility decrements are applied for cancer stage and treatment (largest in year one), survivorship, terminal illness, and false-positive screening. Early-stage cancer decrements combine treatment-specific utility losses [46, 47] with English treatment distributions [23]. We also incorporate reductions in quality of life associated with later-stage breast cancer, longer-term survivorship, end-of-life care and a UK-specific disutility for false-positive screening [48–50].

### Cost-utility analysis

We conduct a cost-utility analysis, with results expressed as net monetary benefit (NMB) to capture the value of each intervention in monetary terms while accounting for the opportunity cost of service change to the NHS [51]. In accordance with NICE 2025 guidance [18], NMBs are calculated at a willingness-to-pay threshold of £20,000 per QALY. When comparing multiple interventions, the option with the highest NMB at the threshold is considered most cost-effective. Strategies that reduce costs while improving health outcomes compared with standard screening are termed dominant [51]. Incremental cost-effectiveness ratios (ICERs), which express the additional cost per additional QALY gained, are reported for strategies that are not dominant relative to standard screening.

### External validation and sensitivity analyses

External validation of the model was carried out using benchmarks from 2022 NHS breast screening data [23] (appendix, pp 41–42). Probabilistic sensitivity analysis (PSA) used parameter distributions detailed in the model documentation (appendix, pp 49-53). To minimise stochastic error, each run simulated 100,000 individuals, with 2000 PSA iterations balancing precision and computational feasibility. The adequacy of the number of PSA runs was assessed by reviewing the variance in NMB, as recommended by NICE guidance [18], to ensure that increasing the number of iterations would not substantially affect the results (appendix, pp. 49-50). Model uncertainty was also assessed with scatterplots and cost-effectiveness acceptability curves (CEACs), showing the probability each strategy is most cost-effective across willingness-to-pay thresholds [51]. An additional CEAC is presented for a scenario excluding the most cost-effective AI strategy, as its feasibility to policymakers is not known, thereby allowing a clearer comparison of how the alternative strategies perform in terms of cost-effectiveness. One-way deterministic sensitivity analyses incrementally increased the cost of AI per screen by up to double.

## Results

In validation testing, the model’s performance under standard screening predicted the proportion of tumours detected as DCIS to be 14.9%, closely matching the empirical estimate of 13.8% from national audit data. The model estimated that 51.7% of cancers would be screen-detected, compared with an observed rate of 46.2%. This modest difference with observed data falls within the expected variation for population-level models.

Table 1 presents the base-case economic results, showing the lifetime economic impact per woman in the cohort (women invited to screening at age 50). All AI-based strategies were associated with increased QALYs compared to the current screening programme. Replacing both human readers with AI generated cost savings of £160 per woman and produced the highest net monetary benefit, at £284,319 per woman, using a £20,000 per-QALY threshold. Replacing one human reader with AI also resulted in cost savings (£31 per woman) and produced the second-highest net monetary benefit (£284,193). In contrast, the strategy with two human readers plus AI (triple reading) generated the largest QALY gain (0.00023, compared with 0.00019 for one human reader plus AI and 0.00009 for AI-only reading), but it also incurred additional costs of £72.79 per woman. This strategy therefore had the lowest NMB (£284,090) and an ICER well above the £20,000 threshold (£317,423), making it not cost-effective compared to all screening alternatives. By contrast, the other AI strategies are dominant, providing both cost savings and health improvements over standard screening. In deterministic sensitivity analysis (appendix, pp. 42-44), a 100% increase in AI costs reduced the cost savings of the AI-only strategy, but it maintained its position of having the highest NMB (£288,013 per person, at the £20,000 per QALY threshold).

**Table 1:** Health and cost outcomes, incremental effects, and NMB of screening strategies.

Screening strategy	Outcomes per person invited		Screening strategy vs. standard care		Percentage change vs. standard care		ICER (vs. standard care)	NMB (£)
	QALYs	Costs (£)	QALYs (95% CI)	Costs (£) (95% CI)	QALY (%)	Costs (%)	Cost per QALY (£)	£20k cost per QALY
Standard care	14.59128	7668	-	-	-	-	-	£284,158
Double reading (1 human + AI)	14.59147	7637	0.00019 (0.00015, (0.00022)	−31.07 (−32.05, −30.10)	0.0013	−0.41	Dominates standard screening	£284,193
Triple reading (2 humans + AI)	14.59151	7741	0.00023 (0.00019, (0.00027)	72.79 (71.82, 73.75)	0.0016	0.95	£317,423	£284,090
Single reading (AI only)	14.59137	7508	0.00009 (−0.00077, 0.00094	−159.55 (− 160.83, −158.27)	0.0006	−2.08	Dominates standard screening	£284,319

Table 2 presents the clinical and screening outcomes for a cohort of 100,000 women invited to screening at age 50. Cancer outcomes are reported for the first diagnosed cancer, distinguishing these from recurrences. All AI-based strategies demonstrated improvements compared to the current screening programme. Compared to standard screening, double reading with one human and one AI detected more cancers through screening (4840 vs 4621), decreased the proportion of advanced cancer cases (11.9% vs 12.2%) and reduced cancer-related deaths (1506 vs 1545). The triple reading strategy (two human readers plus AI) produced the highest overall number of screen-detected cancers (4860), the smallest proportion of advanced cancer cases (11.6%) and the fewest cancer deaths (1491). Single reading with AI resulted in more cancer deaths (*n* = 1,535) than the other AI-based strategies, but still fewer than standard screening (1545). It detected a slightly higher proportion of cancers through screening (52.7% vs 51.7% under standard screening) and marginally reduced the proportion of advanced cancers (12.0% vs 12.2%).

**Table 2:** Cancer detection, mortality, and years of life by screening strategy.

Screening strategy	Initial cancers detected by stage, No. (%)			Cancer deaths, No. (%)	Screen-detected cancers, No. (%)
	DCIS	Stages I-II	Stages III-IV		
Standard care	1295 (14.5)	6557 (73.3)	1089 (12.2)	1545 (17.3)	4621 (51.7)
Double reading (1 human + AI)	1333 (14.9)	6542 (73.2)	1066 (11.9)	1506 (16.8)	4840 (54.1)
Triple reading (2 humans + AI)	1323 (14.8)	6584 (73.6)	1034 (11.6)	1491 (16.7)	4860 (54.4)
Single reading (AI only)	1276 (14.3)	6595 (73.8)	1070 (12.0)	1535 (17.2)	4698 (52.5)

**Fig. 3:**
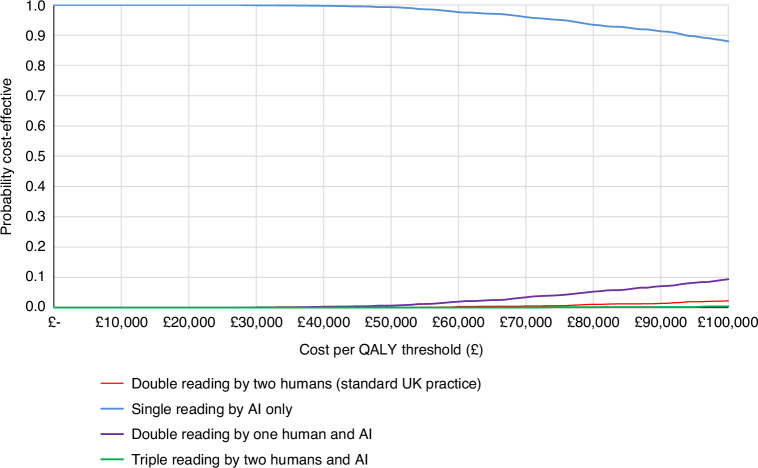
Cost-effectiveness acceptability curve for screening strategies. The figure shows the probability that each screening strategy is cost-effective across a range of cost-per-QALY thresholds. Strategies shown are double reading by two humans, single reading by AI only, double reading by one human and AI, and triple reading by two humans and AI.

Probabilistic uncertainty is illustrated in the scatterplots (appendix, pp. 45–46) and CEAC (Fig. 3). The AI-only strategy has a 100% probability of being most cost-effective at a £20,000 threshold and a 99.9% probability at £30,000. When this strategy is excluded from consideration, the single human reader plus AI strategy becomes the most cost-effective option, with an 88.0% probability of being most cost-effective at £20,000 and an 84.7% probability at £30,000 (appendix, pp. 48–49).

## Discussion

We modelled a cohort of 100,000 women invited to breast screening at age 50, capturing interval cancers as well as the long-term prognosis of all women in the cohort. We find that replacing both human readers with AI in breast cancer screening is highly likely to be the most cost-effective strategy. Replacing one human reader with AI is also cost-effective compared with standard screening. In contrast, the double-reading strategy using two human readers plus AI is not cost-effective, despite producing the largest QALY gain per woman.

The modest gains in NMB observed for the AI strategies are attributable to two factors. First, the improvements in diagnostic accuracy are relatively small and may even be attenuated when implemented in routine UK clinical practice. Second, the benefits are expressed per woman invited to screening, rather than per attendee, which dilutes the health gains from screening. Extrapolating the per-woman benefits to the 163,016 first-time invitees in 2024 [52] shows that AI-only reading provides an annual net benefit of £26.29 million at a £20,000/QALY threshold and saves the NHS £26.01 million per year compared with current practice. In the context of the NHS breast screening programme’s 2024/25 expenditure of £199.47 million [53], these savings represent a comparatively modest reduction in overall programme costs. Moreover, caution is warranted when interpreting these cost-saving estimates, as they rely on scaling outcomes from a single cohort entering the programme at age 50. The NHS Breast Screening Programme comprises multiple overlapping cohorts aged 50–71 [6], each at different stages of the screening pathway. A multi-cohort model that simulates all age groups simultaneously would likely give a different estimate of the total annual population-level impact on NHS costs, although the relative ranking of strategies in terms of cost-effectiveness would be expected to remain unchanged.

A key strength of this study is that our diagnostic accuracy parameters were derived from a validated AI system, which was prospectively tested in the Swedish breast screening programme through a large, population-based clinical trial [17]. We deliberately chose Swedish data because their screening practices closely resemble those of the UK, including training, technology, quality assurance, and population demographics [54]. As there are currently no prospective data comparing AI in breast cancer screening in the UK, the ScreenTrustCAD trial provides a high-quality and methodologically rigorous source. Although the UK screens every three years and Sweden every two, the strong similarities between programmes support the generalisability of these findings to the UK.

A strength of this study is the use of a natural history model that captures tumour progression, interval cancers, and lead-time effects, enabling a realistic representation of screening outcomes. Another strength is that this is the first UK breast cancer screening model to explicitly account for recurrence, linking both the incidence and duration of recurrence to the cancer stage at initial diagnosis. This allows for a more accurate assessment of the long-term health and economic benefits of screening. Our model also incorporates transitions to surveillance (annual screening) after cancer detection, as well as detailed screening attendance patterns that reflect prior attendance, age, and invitation history. This approach of closely replicating real-world pathways and attendance behaviour is necessary for accurately modelling interval cancers and for quantifying the added value of screening in reducing their occurrence.

This study has several limitations that should be considered when interpreting the findings. Firstly, the AI licensing costs included in our analysis were obtained from a UK regulatory authority [38]. However, these costs relate to a different AI system than the one evaluated in the Swedish diagnostic accuracy study [17], as no published costs are available for the specific system used in that trial. This introduces some uncertainty regarding the true per-screen cost of AI. However, our sensitivity analysis showed that even when all AI costs were doubled, the strategy of AI replacing a human reader remained cost-effective.

The model assumes that AI-detected cancers share the same stage distribution, and consequently the same overdiagnosis rate, as cancers identified through standard screening. In reality, AI may shift the distribution of stages at detection or identify indolent lesions that would not otherwise become symptomatic. Additional research is therefore required to establish whether AI affects overdiagnosis rates, as this would have important implications for treatment costs and long-term outcomes [9]. In addition, potential effects of AI on patient attendance, satisfaction, or anxiety were not modelled, and we assumed no change in participation rates with the introduction of AI. Finally, the analysis does not address possible changes in AI performance over time due to software updates or exposure to new data. If AI accuracy were to improve, the cost-effectiveness of this approach would increase further.

A limitation of the analysis is that a full evaluation of breast screening policy would ideally include a no-screening baseline to estimate the absolute benefits of triennial mammography. The present study was designed to compare alternative reading strategies within the existing NHS Breast Screening Programme, and the model was therefore not structured to simulate a no-screening counterfactual. Nonetheless, the broader question remains relevant for policy because the value of investing in AI-based screening ultimately depends on the underlying cost-effectiveness of the screening programme itself.

Our findings are consistent with recent economic models. Vargas-Palacios and colleagues [15] simulated the NHS breast screening programme and found that replacing one human reader with AI could be cost-effective if accuracy and costs are acceptable, but their analysis relied on retrospective data and did not account for interval cancers or detailed clinical pathways. Hill and colleagues [30, 31] modelled AI-guided risk stratification, showing potential QALY gains and cost savings, but focused on personalised screening intervals rather than direct AI-based cancer detection.

Our research is especially relevant as the NHS accelerates efforts to evaluate AI in cancer screening [55], supported by major investment in large-scale, real-world evaluation. The newly launched £11 million NIHR-funded EDITH trial will assess the impact of AI in breast cancer screening across nearly 700,000 women at 30 UK sites [56]. As real-world evidence emerges, ongoing evaluation and monitoring will be essential to ensure AI is deployed safely, effectively, and equitably.

The modelling results indicate that AI-based screening is highly cost-effective, but translating these benefits into routine NHS practice will depend on several practical, regulatory, and organisational factors [57]. Implementing AI at scale requires compliance with evolving medical-device regulations and data-governance frameworks, including clear processes for performance monitoring. Strong data-privacy and cybersecurity protections are also essential, given the vulnerability of large interconnected digital systems. Professional acceptance and patient trust will further shape adoption, especially where AI replaces or substantially reduces human oversight [58]. Together, these considerations may influence both the pace of implementation and the extent to which the cost-effectiveness estimated in this study is realised in real-world NHS settings.

By drawing upon prospective clinical trial data, our study provides robust real-world evidence on the cost-effectiveness of AI in cancer detection and its impact across the screening pathway. Future evaluations should also consider the combined use of AI tools for both cancer detection and risk prediction, as these processes could feasibly be integrated within a single AI assessment. Evidence from risk-stratified models suggests that targeting high-risk individuals with more frequent monitoring could lead to health improvement without additional NHS costs [30, 31] and while maintaining overall screening levels [59]. Beyond potential cost savings, AI technologies could also reduce reliance on radiographers, accelerate diagnosis, increase productivity, reduce waiting times, and improve screening accuracy [14], all of which are key priorities under the recently published NHS Long Term Plan [55]. Although these advantages are compelling, they remain largely theoretical, and real-world evidence from UK implementation studies will be essential to establish their feasibility and impact within a publicly funded screening system. In light of this uncertainty, investment decisions should balance the potential advantages of AI with a rigorous assessment of opportunity costs and the equity implications of modifying the current screening pathway [60].

## Supplementary information

Appendix

## Data Availability

Authors can confirm that all relevant data are included in the paper or supplementary appendix. All data used to populate the model is publicly available and referenced. All parameters and their values are in the appendix.
