# Supplementary material for: Economic evaluation of artificial intelligence for cancer detection in the UK breast screening programme
Source: Br J Cancer. 2026 May 2;135(3):453–60. doi: 10.1038/s41416-026-03465-3 (PMC13373195; doi:10.1038/s41416-026-03465-3)
Supplement: Supplementary file 1 — Appendix [file 41416_2026_3465_MOESM1_ESM.docx]

**APPENDIX**

This appendix provides technical data, outlines the methodological approaches used, and lists the reference materials supporting this work.

**Breast Screening Attendance**

This appendix sections outlines the approach for simulating breast screening attendance within the model. The methodology reflects both national screening data and individual-level behavioural reinforcement observed in prospective studies.

**Invitation Intervals and Cessation of Attendance**

Eligibility for breast screening invitations is determined by both age and the prescribed interval between screenings. Women typically receive routine invitations every three years, beginning at age 50 and ending at age 71. In contrast, women undergoing surveillance, such as those with a history of cancer diagnosis, are invited for annual screenings for a period of ten years. Upon completion of this surveillance period, if they remain eligible (under the age of 71), they revert to the standard three-year invitation cycle.

Invitation to screening ceases under specific circumstances: if an individual surpasses the upper age threshold of 71 years or if the individual has died. Attendance at screening is modelled probabilistically. For each invitation, a random value is drawn and compared to the individual’s calculated probability of attendance. If the random value falls below this threshold, the individual is recorded as having attended; otherwise, non-attendance is recorded. The probability is influenced by several factors including whether it is their first screen, whether they experienced a false positive at their last screen and the number of consecutive previous attendances (the “reinforcement streak). Should an individual decline an invitation, their “reinforcement streak” is reset, and their future attendance probability reverts to that associated with non-consecutive attenders within their age group.

**Baseline Probabilities of Attendance by Age and Screening History**

At each eligible invitation, every simulated individual is assigned a probability of attending based on their age and their history of screening invitations and attendance. These probabilities are drawn from National Breast Cancer screening Audit data from 2022 to 2023 (NHS Digital, 2024). The model applied these probabilities depending on whether the invitation is the individual’s first, a re-invitation to a never-attender, a subsequent screen after a recent attendance (last screen in 5 or less years), or after a longer lapse (more than five years) as summarised in Table A1.

Table A1: Age- and Context-Specific Invitation Uptake (England, 2022–23)

| **Age band (years)** | **First-ever invite** | **Never attended** | **Subsequent (≤5y since last)** | **Subsequent (>5y since last)** |
| --- | --- | --- | --- | --- |
| 45–49 | 0.57 | 0.241 | 0.738 | 0.358 |
| 50–52 | 0.588 | 0.272 | 0.767 | 0.547 |
| 53–54 | 0.338 | 0.211 | 0.78 | 0.511 |
| 55–59 | 0.288 | 0.123 | 0.799 | 0.452 |
| 60–64 | 0.291 | 0.074 | 0.821 | 0.407 |
| 65–70 | 0.28 | 0.05 | 0.841 | 0.367 |
| Over 70 | 0.28 | 0.056 | 0.822 | 0.351 |

These probabilities are the base data used for deriving attendance after consecutive attended screens and after a false positive.

**Reinforcement of Attendance After Consecutive Screens**

Attendance probability increases after consecutive past attendance. This “reinforcement effect” is derived from the UK breast screen randomised controlled trial, the Age Trial (Johns and Moss, 2010), which reported cumulative uptake for each sequential invitation among women who had attended all previous screens. The model applies a multiplicative uplift in Table A2 to the age-based probability for individuals who attended screening within the last five years.

These multipliers are only applied up to the observed ceiling in attendance percentage, as reported in the Age UK trial (Johns and Moss, 2010) which is 92.6% at the 8th and subsequent screens. The multipliers, reported in Table A2, are not compounded if an individual misses a screen. If the attendance streak is broken, the reinforcement resets.

Table A2: Multiplicative Reinforcement Weights for Successive Attendance

| **Number of successive attendances** | **Reinforcement multiplier** |
| --- | --- |
| 2 (third invitation) | 1.069 |
| 3 (fourth invitation) | 1.068 |
| 4 (fifth invitation) | 1.035 |
| 5 (sixth invitation) | 1.036 |
| 6 (seventh invitation) | 1.029 |
| ≥7 (eighth+ invitation) | 1.014 |

**Attendance Penalty After a False Positive Result**

Attendance probability is reduced for the screen immediately following a false positive (FP) result. The effect is modelled as a one-time penalty using hazard ratios derived from meta-analyses of studies examining breast screening attendance. Specifically, it is the ratio attendance after a false positive (60%) to that of normal attendance (68%) in a meta analysis of studies reporting factors associated with attendance at screening for breast cancer (Mottram et al., 2021). The model applies this ratio (0.88) as a multiplicative down lift to the age-based probability for individuals who attended screening within the last five years in Table A1. This adjustment is applied as a single effect; the probability returns to its original value for subsequent invitations.

**Mortality**

For each individual in the cohort, age at death was simulated using a discrete year-by-year sampling algorithm based on recent (data for the years 2021-2023) national life table mortality rates for adult women in England (ONS, 2025). The simulation began at a defined model entry age (e.g., 50 years), using only the life table rows at or above this age. For each individual, a random number between 0 and 1 was drawn for every subsequent age. At each year, if the random number was less than the corresponding age-specific probability of death, the individual was assigned that age as their age at death, and simulation for that individual stopped. If an individual did not meet this criterion at any age (i.e., they survived through the maximum age in the table), their age at death was set to 99 years. This process yields a realistic distribution of ages at death for the simulated population, fully reflecting the year-by-year mortality risks specified in the national life tables.

**Natural History Of Cancer**

The natural history sub-model forms the foundation for simulating each woman’s breast cancer trajectory within the economic analysis. Each woman’s breast cancer journey is simulated from initial onset through to symptomatic detection, and post detection recurrence. An illustration is in Figure 1 in the main manuscript.

For each simulated individual, the model established a set of natural history variables prior to when the model is run: cancer status (whether a case of cancer between the ages of 50 and 74 in the model), age at cancer onset (for cancer onset), recurrence cancer incidence (by initial cancer stage), and times to recurrence for both invasive cancers and DCIS initial cancers. These aspects of the model are drawn from the highest quality available epidemiological evidence: recent meta-analyses, large population studies, and national cancer registries.

**Assigning Cancer Status**

At the start of the simulation, each individual is assigned a cancer status, whether or not they develop breast cancer during the simulation, based on a 9.05% probability of developing breast cancer in women in England from age 50 to 74. This approach ensures that the simulated incidence rates accurately reflect cases that could potentially be detected through the national screening programme, which screens individuals from ages 50 to 70. By allowing cancer incidence up to age 74, three years beyond the final screening age, we account for cancers that may have been present but undetected at the last screening appointment (at age 70) and only become symptomatic later (we assume up to three years later). Age 74 is chosen as the cutoff because the available incidence data is grouped by age bands, with the relevant band being 70–74. This method is intended to capture all cases that existed during the screening period, even if they did not emerge or were not detected until after screening ended at age 70. The probability of 9.05% is taken from Cancer Registration Statistics in England (NHS Digital, 2024) and represents cases of malignant breast cancer (registry data classification code: ICD-10 C50) and carcinoma in situ of the breast (registry data classification code: ICD-10 D05).

**Age at Symptomatic Detection**

Each simulated individual is assigned an age at which their breast cancer progresses to the point of symptomatic detection, meaning the cancer is discovered due to symptoms during a primary care visit rather than at a scheduled screening appointment. Symptomatic cancers, in this context, are detected in the intervals between scheduled screening. The age of symptomatic detection is thus the latest possible age at which a cancer could initially be found, and the modelled benefit of screening is that it identifies these cancers at earlier ages, usually at an earlier stage.

To assign the age at symptomatic detection, the model uses age-specific breast cancer incidence rates derived from the Cancer Registration Statistics for England, specifically for women aged 50 to 74, to align with data used for the incidence probability of 905%. These are reported in Table A3. For each simulated woman, the model first determines whether her cancer would be detected above the upper screening age of 70. This is based on the observed proportion of incident cancers in women aged 70–74, which is 16.4%. If a randomly drawn value is less than or equal to this probability, her age at symptomatic detection is set randomly between ages 71 and 74. If the draw is higher, the woman is assigned an age within the screening programme range, and the process then samples from the distribution of screen-detected cancers in the UK programme taken from screening programme data (NHS Digital, 2025). This distribution is show in Table A4. If the sample detection age exceeds the woman’s age of all-cause death, the detection age is resampled to ensure that no individual is assigned a cancer detection age after their death.

Once the relevant age band is determined, a uniform distribution is used to select an exact age within that band. However, the ages at which cancers are detected through screening differ from those at which they would present symptomatically, therefore an additional adjustment is applied for individuals whose sampled detection ages fall between 50 and 70 (that is, those assigned from the screen-detected cancer age distribution). To estimate the age at which cancer would be detected symptomatically, the model adds a fixed delay of 0.8 years to the screen-detected age. This value reflects the average difference observed in a recent empirical study of the English breast screening programme (Bhatt., 2024). In this study the mean age for women with breast cancer clinically detected was 50.8 years, and 58.0 years for screen detection. The following tables are the age-group probabilities, derived from national data, used in this sampling process.

Table A3. Age distribution of general cancer-incidence

| **Age group** | **Probability** |
| --- | --- |
| 50 – 54 | 0.231 |
| 55 – 59 | 0.231 |
| 60 – 64 | 0.202 |
| 65 – 69 | 0.171 |
| 70 – 74 | 0.164 |

Table A4. Age distribution of screen-detected breast cancer

| **Age group** | **Probability** |
| --- | --- |
| 50 – 54 | 0.223 |
| 55 – 59 | 0.22 |
| 60 – 64 | 0.252 |
| 65 – 70 | 0.305 |

**Age of Tumour Onset**

After assigning the age at which symptomatic detection occurs, the model estimates the age at which a tumour could first be identified through screening, prior to the onset of symptoms. This is done by sampling from a normal distribution, with the mean and standard deviation of the tumour preclinical phase estimates using data from Isheden and Humphreys (2019). This source is chosen because it provides the most recent modelling estimates based on a dedicated natural history model of breast cancer, and because its estimates are specific to the individual’s age at symptomatic detection. For women aged 50 to 74, the average preclinical phase across the population is 7.13 years. Back calculation is then used to find the true onset age: symptomatic detection age minus the sampled value of preclinical duration. This approach produces a heterogeneous distribution of cancer onset ages.

**Modelling Recurrence**

The model goes beyond initial diagnosis by projecting each woman’s risk recurrence. For invasive cancers, recurrence is tracked for up to 25 years, with risk curves tailored by stage (1, 2–3, 4). For DCIS (ductal carcinoma in situ), a distinct annual risk curve is used for up to 20 years. These curves are sourced from the latest large-scale cohort studies and clinical trials, ensuring validity and applicability.

Before running the simulation, each woman with cancer is pre-assigned whether a recurrence will occur, along with a time interval from the initial cancer to recurrence for every possible stage of initial cancer. Then, based on the initial cancer stage, the model refers to the pre-assigned values for that stag to determine if and when a recurrence happens. The stage of recurrence is determined using the same process as the initial stage assignment, with the additional step of resampling if the assigned stage is lower than the initial cancer stage. Epidemiological evidence supports this assumption. Chua et al., 2024) has demonstrated higher stage and grade were associated with recurrence in women with early-stage estrogen receptor–positive breast cancer. A recent meta-analysis has found that breast cancer patients diagnosed initially at higher stages (IIb–IIIc) are more likely to experience metastatic recurrence than those whose initial cancer was earlier stage (Morgan et al., 2024). An epidemiological study has found that cancer initially diagnosed at a higher stage (e.g., Stage III) continue to have worse survival even after recurrence, demonstrating that recurring cancer is not downgraded in severity (Hasset et al., 2017).

The annual risk of recurrence over twenty years for women diagnosed with DCIS through screening in England is based on the absolute risks reported in Mannu et al. (2020), which analysed over 35,000 women diagnosed with DCIS. For invasive cancers, recurrence risks are modelled separately by cancer stage at diagnosis (stages 1, 2/3, and 4), with annual probabilities up to 25 years taken from Colleoni et al. (2016). This long-term recurrence data by invasive stage comes from the International Breast Cancer Study Group Trials, which are considered among the highest quality sources of evidence. The DCIS and invasive cancer recurrence risks are shown in Table A5.

Table A5. Annual absolute risk of recurrence by initial cancer stage.

| **Year since initial cancer diagnosis** | **DCIS Absolute Risk (%)** | **Stage 1 (%)** | **Stage 2/3 (%)** | **Stage 4 (%)** |
| --- | --- | --- | --- | --- |
| 1 | 0.3 | 1.77 | 1.14 | 5.32 |
| 2 | 0.3 | 1.77 | 1.14 | 5.32 |
| 3 | 0.4 | 1.77 | 1.14 | 5.32 |
| 4 | 0.3 | 1.77 | 1.14 | 5.32 |
| 5 | 0.3 | 1.77 | 1.14 | 5.32 |
| 6 | 0.4 | 0.48 | 0.25 | 2.07 |
| 7 | 0.3 | 0.48 | 0.25 | 2.07 |
| 8 | 0.4 | 0.48 | 0.25 | 2.07 |
| 9 | 0.3 | 0.48 | 0.25 | 2.07 |
| 10 | 0.3 | 0.48 | 0.25 | 2.07 |
| 11 | 0.4 | 0.14 | 0.15 | 0.8 |
| 12 | 0.3 | 0.14 | 0.15 | 0.8 |
| 13 | 0.3 | 0.14 | 0.15 | 0.8 |
| 14 | 0.1 | 0.14 | 0.15 | 0.8 |
| 15 | 0.3 | 0.14 | 0.15 | 0.8 |
| 16 | 0.3 | 0.12 | 0.06 | 0.62 |
| 17 | 0.3 | 0.12 | 0.06 | 0.62 |
| 18 | 0.3 | 0.12 | 0.06 | 0.62 |
| 19 | 0.3 | 0.12 | 0.06 | 0.62 |
| 20 | 0.3 | 0.12 | 0.06 | 0.62 |
| 21 | - | 0.02 | 0.04 | 0.27 |
| 22 | - | 0.02 | 0.04 | 0.27 |
| 23 | - | 0.02 | 0.04 | 0.27 |
| 24 | - | 0.02 | 0.04 | 0.27 |
| 25 | - | 0.02 | 0.04 | 0.27 |

In summary, the structure of establishing the natural history of cancer of the model is designed to reflect the best evidence on incidence, age of tumour onset, age of symptomatic detection, recurrence incidence, and time from first cancer to any such recurrence. Together, these outputs capture the natural history of breast cancer, forming the basis for downstream analyses that overlay screening programs onto the simulated disease trajectories to estimate population impact and cost-effectiveness. We now turn to how the stage of cancer is assigned upon detection.

**Stage Distribution Assignment in the Economic Model**

The assignment of stage at diagnosis in the economic model follows a two‐step probabilistic sampling process anchored to real‐world audit data. First, each woman who develops breast cancer is classified into one of eight five‐year age bands (50–54, 55–59, …, 85+). Within her age band, the probability of ductal carcinoma in situ or invasive stages 1–4 is taken from the National Audit of Breast Cancer in Older Patients annual reports. The stage distribution of cancers detected by symptoms includes a greater proportion of advanced cases compared to those found through screening, and in both groups, older women have a higher proportion of advanced cancers.

If a woman experiences a recurrence, a second draw is made using the same age‐banded, screen‐ vs symptomatic–specific distributions. To reflect clinical reality, the model prohibits downgrading at recurrence: the recurrent stage cannot be lower (i.e. numerically smaller) than the original. This ensures that, for example, a primary stage 3 disease cannot recur as stage 2.

**Source Data and Derivation of Age- and Stage-Specific Probabilities**

For cancers detected, the distribution of DCIS and stages 1–4 comes by age and mode of detection is based on two reports with audit data women in England aged 50 years or older with breast cancer (NABCOP, 2020, 2022). There is more recent data on the cancer in the 2022 report but stages 1–3 are unavailable in the later audit. Therefore, the relative change in DCIS and stage 4 frequencies between the 2020 and 2022 reports is applied to the finer 2020 stage and age bands. The relative change of DCIS and stage 4 was calculated separately for screen-detected and symptomatic cancers. This approach, of applying relative changes (from 2020 to 2022) to the 2020 distribution of DCIS and stage 4 is used for two reasons. To have a more recent profile of cancer stages by mode of detection (screen or symptomatic) than in the 2020 report. The 2020 distribution is the base distribution because it reports staging across twice as many number of age bands as the 2022 data. For instance, the age bands used in the 2022 report is 50–69 years while in 2020 it is disaggregated to 50–54 years and 55–59 years. Therefore, replacing the 2020 data entirely with the 2022 data would result in a loss of age information, while the approach we have taken gives a distribution across the 2020 age bands (which is more age bands than 2022)*.* Table A6 is the resulting age‐specific stage distributions used for assignment, with separate columns for screen‐detected and symptomatic detections.

Table A6. Age and mode of cancer detection-based cancer stage distributions

| Age group | Screen DCIS | Screen Stage 1 | Screen Stage 2 | Screen Stage 3 | Screen Stage 4 | Symptomatic DCIS | Symptomatic Stage 1 | Symptomatic Stage 2 | Symptomatic Stage 3 | Symptomatic Stage 4 |
| --- | --- | --- | --- | --- | --- | --- | --- | --- | --- | --- |
| 50–54 | 0.2527 | 0.4687 | 0.2358 | 0.0362 | 0.0065 | 0.0691 | 0.2884 | 0.4598 | 0.1283 | 0.0544 |
| 55–59 | 0.2247 | 0.5117 | 0.224 | 0.0325 | 0.0071 | 0.0589 | 0.2864 | 0.4555 | 0.1307 | 0.0686 |
| 60–64 | 0.1946 | 0.5568 | 0.2124 | 0.0297 | 0.0065 | 0.0579 | 0.2813 | 0.4581 | 0.1265 | 0.0762 |
| 65–69 | 0.1824 | 0.5756 | 0.2097 | 0.0259 | 0.0064 | 0.0557 | 0.2997 | 0.4403 | 0.1172 | 0.0872 |
| 70–74 | 0.1746 | 0.5819 | 0.2084 | 0.0283 | 0.0069 | 0.0518 | 0.2994 | 0.4409 | 0.1154 | 0.0925 |
| 75–79 | 0.153 | 0.5886 | 0.2214 | 0.0323 | 0.0046 | 0.0443 | 0.2797 | 0.4644 | 0.1199 | 0.0917 |
| 80–84 | 0.1518 | 0.5594 | 0.254 | 0.0285 | 0.0064 | 0.0399 | 0.2668 | 0.4706 | 0.1213 | 0.1013 |
| 85+ | 0.1433 | 0.6155 | 0.1894 | 0.0421 | 0.0098 | 0.0387 | 0.2424 | 0.4825 | 0.1348 | 0.1015 |

These probabilities ensure that stage assignment in the model reflects the most recent audit evidence, preserves age‐specific granularity, and accounts for both screen and symptomatic pathways.

**Diagnostic accuracy of the AI Pathways: The Swedish ScreenTrustCAD study**

There are currently no prospective UK studies evaluating the diagnostic accuracy of screening when AI is used either to replace or assist human readers. Therefore, we draw on evidence from a diagnostic accuracy trial conducted in a real-world setting in Sweden. The observed changes screening accuracy from the Swedish paired-reader study are applied to the UK baseline accuracy in routine care (two read mammography) taken from a recent UK clinical trial (Payne et al., 2025). This method incorporates the real-world impact observed in the Swedish trial while ensuring that all projections remain grounded in the actual performance of the UK screening population. The Swedish ScreenTrustCAD study is especially valuable for assessing the relative effect of AI-based interventions on diagnostic accuracy, and in some important respects, it even offers advantages over a conventional randomized trial for this purpose.

First, the trial design was explicitly a prospective, population-based, paired-reader, non-inferiority study. Every mammogram was independently interpreted by both standard human readers and various AI-based workflows within the same screened cohort. This paired comparison eliminates confounding due to case-mix as every case serves as its own control across all interventions, ensuring that any differences in detection are attributable to the AI workflow itself rather than to differences in the underlying population (e.g. cancer risk, cancer incidence, breast density), temporal effects (change in the staffing mix or number of screens), or referral bias (e.g. differences in who is referred to, and who chooses to attend, screening appointments).

Second, the trial was implemented at scale across the entire regional population over a multi-year period, capturing the full spectrum of screening presentations, cancer types, breast densities, and imaging challenges encountered in modern practice. This enhances external validity and ensures that the relative changes estimated are not artifacts of a selected subpopulation, short study window, or simulated test set, but rather reflections of real-world screening conditions.

Third, and most critically, the study’s paired-reader approach provides an exceptionally powerful statistical framework for estimating *relative* diagnostic performance. Because both the AI and the control arms are exposed to the same underlying population, differences in recall, cancer detection, and arbitration rates can be attributed directly to the intervention, independent of the absolute baseline risk or prevalence. This design circumvents many limitations of parallel-arm randomised trials, in which the arms might, despite randomisation, experience subtle differences in case-mix, or where the relatively low prevalence of outcomes can make it difficult to accurately estimate rare-event metrics like interval cancer rate. Moreover, by breaking down accuracy outcomes into recall, arbitration, and outcome ascertainment, the ScreenTrustCAD study directly measures all the key workflow components influencing practical diagnostic accuracy, such as reader disagreement and arbitration. Importantly, the trial reports outcomes by breast density, which is a key factor affecting screening accuracy. This level of detail is especially valuable for modelling the entire screening pathway, as the model does. Therefore, the relative effect sizes from the Swedish study are both statistically valid, due to paired comparisons and a large sample, and broadly generalisable as multiplicative factors (relative changes in accuracy) that can be applied to baseline rates in other populations, like the UK. This makes the ScreenTrustCAD data particularly well-suitedand, and in some ways even preferable to randomized trials lacking these features, when modelling the impact of AI on diagnostic accuracy in a different national context.

**Relative Changes Over Absolute Accuracy in ScreenTrustCAD**

The methodological decision to apply relative changes in screen accuracy to a baseline rather than to use the absolute diagnostic accuracy reported in Swedish ScreenTrustCAD study (Dembrower et al., 2023) is grounded in principles of epidemiological transportability and preservation of real-world program context.

First, the structure of the ScreenTrustCAD trial necessitates the use of relative, not absolute, performance metrics. As detailed in the original publication, the trial’s paired-reader design with screen-positive follow-up ensures that only screen-detected positives, those flagged by either radiologist or AI, are subjected to gold-standard diagnostic work-up. Consequently, while the trial can confidently compare the number of detected cancers and the number of false positive recalls between experimental arms, it cannot determine the absolute number of missed cancers (false negatives) or true negatives in the entire screened population. In other words, while the trial accurately estimates *relative* performance (how much more or less sensitive and specific an intervention is compared to standard double reading), it cannot, by design, provide a population-level estimate of sensitivity or specificity directly applicable to a different program. This justifies the extraction and use of relative true positive fraction (for sensitivity) and relative recall rate for non-cancer cases (for specificity) as the most robust summary measures of diagnostic accuracy impact.

Second, applying these relative measures to a UK basecase confers several critical benefits. The UK NHS Breast Screening Programme differs from the Swedish context in terms of screening intervals, case-mix, population structure (notably in age and breast density distribution), radiologist workflow, and possibly even in the technical characteristics of imaging equipment. Directly importing Swedish sensitivity and specificity values into a UK setting would ignore these structural and epidemiological differences. By instead using UK-specific baseline data, comprising real-world sensitivity and specificity stratified by age and density, the resulting projected outcomes more accurately reflect UK program performance. That is, the AI intervention effects superimposed in a way that preserves the UK screening setting. This method ensures that projections are generalisable to the UK in particular in the downstream outcomes from integrating AI into the programme such as cancers detected, interval cancers, false positives, and resource use.

Below are detailed explanation of how relative changes in diagnostic for AI-based mammography interventions were established, what the values are, and precisely how they are applied in the model.

**UK mammographic sensitivity and specificity**

The baseline performance for sensitivity and specificity by breast density and age is extracted from a recent UK trial (Payne et al., 2025).

This is real-world data reflecting the current, standard practice of double human reading, stratified by Volpara Density Grade and age group (Table A7)

Table A7. Sensitivity and Specificity by Volpara breast density

| **Volpara Grade** | **Age Group** | **Sensitivity (95% CI)** | **Specificity (95% CI)** |
| --- | --- | --- | --- |
| A | ≤60 | 65.4% (47.1–83.7) | 97.3% (96.8–97.7) |
| A | >60 | 81.6% (69.3–93.9) | 98.0% (97.6–98.4) |
| B | ≤60 | 73.1% (65.7–80.4) | 96.0% (95.6–96.3) |
| B | >60 | 73.9% (66.7–81.2) | 97.0% (96.6–97.3) |
| C | ≤60 | 51.8% (42.5–61.2) | 94.7% (94.3–95.2) |
| C | >60 | 70.8% (60.9–80.9) | 96.5% (96.0–97.0) |
| D | ≤60 | 48.1% (34.8–61.5) | 95.1% (94.3–95.8) |
| D | >60 | 58.3% (38.6–78.1) | 97.1% (96.1–98.1) |

**Trial-derived Relative Effects from ScreenTrustCAD trial**

The next step is to extract the relative change in sensitivity (Table A8) and specificity (Table A9) resulting from introduction of AI-based reading strategies. This is directly sourced from Supplementary Table 1 (for sensitivity) and Table 2 (for specificity/recall) of the ScreenTrustCAD Trial (Dembrower et al., 2023).

The relative true positive fraction for cancer cases stratified by intervention and breast density, is applied as the relative sensitivity. The relative recall rate for non-cancer cases interpreted as the relative change in the false positive rate (with specificity being 1 minus this rate). This is extracted from Supplementary Table 1 in Dembrower et al., 2023.

Table A8. Relative Sensitivity (True Positive Fraction)

| **Intervention** | **Volpara Breast Density** | **Mean Relative Increase in Mammography Sensitivity** |
| --- | --- | --- |
| Double-reading by AI + one radiologist | a | 1 |
| Double-reading by AI + one radiologist | b | 1.008475 |
| Double-reading by AI + one radiologist | c+d | 1.098039 |
| Single-reading by AI (used in scenario analysis only) | a | 0.866667 |
| Single-reading by AI (used in scenario analysis only) | b | 0.966102 |
| Single-reading by AI (used in scenario analysis only) | c+d | 1.039216 |
| Triple-reading by two radiologists + AI | a | 1.033333 |
| Triple-reading by two radiologists + AI | b | 1.067797 |
| Triple-reading by two radiologists + AI | c+d | 1.098039 |

*These are ratios: a value >1 means increased sensitivity vs. control; <1 means decreased sensitivity.*

**Relative Specificity (recall rate for people with no cancer)**

The relative false positive rate is extracted from Table 2 in Dembrower et al., 2023, using recall rate for non-cancer cases as a proxy for false positive rate (so, specificity is 1–FP)

Table A9. Relative change in the false positive rate

| **Reading Mode** | **Mean** | **Lower 95% CI** | **Upper 95% CI** |
| --- | --- | --- | --- |
| Double-reading by AI + one radiologist | 0.9391 | 0.92 | 0.96 |
| Single-reading by AI (used in scenario analysis only) | 0.446 | 0.42 | 0.48 |
| Triple-reading by two radiologists + AI | 1.0493 | 1.04 | 1.06 |

*Values <1 mean a reduction in recall/no-cancer (fewer FPs, higher specificity), >1 mean increased recall (lower specificity).*

**How the Relative Effects Are Applied**

For each breast density sub category of women in the model, defined by breast density or age, the projected sensitivity and specificity after introducing AI are estimated by adjusting the baseline real-world values using the relative changes observed in the trial. Sensitivity is updated by applying the trial's average relative change in sensitivity to the baseline. The false positive rate for recall (among those without cancer) is adjusted similarly, using the trial's average change in recall rate. Specificity is then calculated as one minus the projected false positive rate. Example Calculation:

A women’s Volpara breast density category is b, and her age is under 60 years, then the baseline sensitivity is 73.1%. The sensitivity with a human reader and AI read (AI Arm 1) is a mean relative increase = 1.098. That is, the Projected sensitivity = 0.731 × 1.098 = 0.803 (80.3%).

**Comprehensive Breakdown of Cost per Screen Calculation**

This sub section of the appendix details the derivation of all cost-per-screen estimates in our economic model. It provides explanations, sources, calculations, and assumptions for transparency.

**Annual Pay Rates (Salaries)**

Annual salary estimates for staff involved in breast screening were derived from nationally published NHS pay scales for 2023/24. For radiographers, the NHS Agenda for Change (AfC) pay bands were used, while consultant radiologist salaries were based on the British Medical Association (BMA) consultant contract rates. To accurately reflect salary costs across England, adjustments for regional supplements and workforce distribution were applied.

**Regional Salary Weighting**

NHS pay structures include higher rates for posts based in London, reflecting the statutory “High-Cost Area Supplement”: 20% for AfC staff and 15% for consultants. Salary estimates were therefore calculated separately for London and non-London posts. National data from a 2016 survey of the breast imaging workforce reported that 23.05% of breast screening posts were located in London and 76.95% elsewhere in England. These proportions were used to derive a location-weighted national average salary for each staff group.

**Radiographer Salary Estimation**

Radiographers in breast screening services are typically graded between AfC Bands 5 and 7. Median pay points from the 2023/24 AfC scale were selected for each band. The London supplement was added to generate location-specific salary lines. A summary of this information is provided in Table A10. To reflect the staff mix, a weighted average was calculated using an assumed distribution of 60% Band 5, 30% Band 6, and 10% Band 7. This produced an average annual salary of £46,664 for London-based radiographers and £39,699 for those elsewhere in England. Applying the national workforce split, the resulting location-weighted national average radiographer salary was £45,600 per year.

Table A10. Radiographer Annual Salaries by Band and Region

| AfC Band | Median Base Salary (£) | London Supplement | London Total (£) | Non-London Total (£) |
| --- | --- | --- | --- | --- |
| 5 | 34,824 | 20% | 41,789 | 34,824 |
| 6 | 42,636 | 20% | 51,163 | 42,636 |
| 7 | 51,640 | 20% | 61,968 | 51,640 |

*Grade-weighted averages: £46,664 (London), £39,699 (outside London).*
*Location-weighted national average: £45,600.*

**Consultant Radiologist Salary Estimation**

Consultant radiologist salaries were calculated using the BMA contract rates for the three most common spine points (MC75–MC77) in 2023. London salaries were derived by applying the 15% high-cost supplement. A simple average across the three points provided mean values for London (£124,961) and for the rest of England (£108,082). Applying the 23.05%/76.95% workforce split yielded a location-weighted national average consultant salary of £113,962 per year. This information is summarised in Table A11.

Table A11. Consultant Radiologist Annual Salaries by Spine Point and Region

| Spine Point | National Base Salary (£) | London Supplement | London Total (£) | Non-London Total (£) |
| --- | --- | --- | --- | --- |
| MC75 | 99,532 | 15% | 114,462 | 99,532 |
| MC76 | 105,390 | 15% | 121,199 | 105,390 |
| MC77 | 119,323 | 15% | 137,221 | 119,323 |

*Simple average: £124,961 (London), £108,082 (outside London).*
*Location-weighted national average: £113,962.*

**Regional distribution of the screening workforce**

The workforce distribution used for salary weighting is summarised in Table A12, from published national survey data (British Society of Breast Radiology, 2016).

Table A12. Workforce regional distribution of breast screening readers.

| Region | Posts | Share of Total (%) |
| --- | --- | --- |
| England (non-London) | 247 | 76.95 |
| London | 74 | 23.05 |
| Total | 321 | 100 |

All annual salary estimates reported above are based on officially published pay scales, with region and grade distribution chosen to closely reflect the structure of the NHS breast screening workforce. These location- and grade-weighted salaries serve as direct inputs to national microcosting and cost-effectiveness analysis. Per-screen reading costs are subsequently derived from these salaries by dividing by empirically observed annual workloads (3,618 mammograms per year for radiographers and 3,411 for consultant radiologists, as reported by Chen et al., 2023). This approach ensures that the staff cost components of the economic evaluation are representative of both the pay structure and actual deployment of NHS staff in England.

**Staff Pay Costs per Screen**

**Workforce Composition and Volumes**

The NHS Breast Screening Programme (NHSBSP) relies on a double-reading protocol, in which each mammogram is independently reviewed by two readers. One reader is typically a consultant radiologist and the other a trained radiographer. To estimate the cost per screen, we begin by establishing the annual reading volumes for each profession.

Workforce counts are based on the latest available data (Chen et al. 2023), and annual read volumes come from the same national audit of the NHSBSP. In that retrospective study, 224 radiologists and 177 radiographers collectively double-read about 1.404 million screening mammograms in England, with radiologists reading 763,958 and radiographers 640,437 first-read exams. Dividing total reads by staff count yields gave an average annual workload per reader of 3,411 reads for each radiologist and 3,618 reads for each radiographer. These values form the basis for converting annual salary costs for radiologists and for radiographers into a cost per individual reader in our model.

To calculate the cost of a typical screening mammogram read, we do not model radiologists and radiographers as separate categories within the routine double-reading pathway. Instead, we construct a single composite reader, representing a weighted blend of the two professions based on their real-world contribution to the reading workload. This composite is derived by proportionally averaging the cost per read of each profession (as determined by dividing salary by annual workload), weighted by the observed share of total reads (1,404,395) performed by each group, 54.4% by radiologists and 45.6% by radiographers, based on the NHSBSP audit (Chen et al. 2023). The result is a representative cost per read, which reflects the actual composition and cost of the NHS breast screening reading workforce. This weighted-average "composite" reader is then used as the cost basis for all reader interpretation of a breast screen in the model. This composite approach is necessary because, in practice, it is unclear which specific staff group (radiologists or radiographers) would be replaced if artificial intelligence were introduced to reduce human workload. As a conservative and neutral assumption, the model therefore presumes that AI displaces both groups in proportion to their existing workload i.e., radiographers and radiologists are replaced in equal measure, according to the observed 45.6% / 54.4% split in reader volumes. Notably, the audit found no difference in cancer detection performance between the two groups, reinforcing the validity of treating their reads as equivalent in the cost model. By weighting each group’s cost per read accordingly, we derive a composite cost per read of £25.10. We apply this £25.10 figure as the representative cost of one reading event in the current system. With this composite cost per read, the model can directly estimate the change in staff-pay costs resulting from the removal of a human reader under different AI integration scenarios.

This approach relies on accurate estimation of the average annual salary costs for radiologists and radiographers. To derive these, we undertook detailed micro-costing of their salary, factoring in standard salaries, the distribution of staff across different experience levels and the corresponding salary progression associated with seniority, regional London weightings, and the additional uplift for locum usage.

**Staff Pay Rates (Salaries)**

All annual salary estimates reported below are based on officially published pay scales, with region and grade distribution chosen to closely reflect the structure of the NHS breast screening workforce. Per-screen reading costs are subsequently derived from these salaries by dividing by empirically observed annual workloads (3,618 mammograms per year for radiographers and 3,411 for consultant radiologists, as reported by Chen et al., 2023). This approach ensures that the staff cost components of the economic evaluation are representative of both the pay structure and actual deployment of NHS staff in England.

**Radiographer Salary Estimation**

Radiographers are job roles graded between AfC Bands 5 and 7. The median pay point within Band 7 was used because only advanced practitioner radiographers, a job role at AfC grade 7, are allowed to read within the NHS Breast Screening Programme. NHS pay structures include higher rates for posts based in London, reflecting the statutory “High-Cost Area Supplement”:  inner London (+20%); outer London (+15%); and fringe London (+5%). Salary estimates were therefore calculated separately for London and non-London posts, assuming the London supplement to be an average of the inner, outer and fringe supplements. National data from a 2016 survey (British Society of Breast Radiology, 2016) of the breast imaging workforce reported that 23.05% of radiographer breast screening posts were located in London and 76.95% elsewhere in England. These proportions were used to calculate a location-weighted national average salary of breast screening radiographer in England of £42,505 per year.

**Consultant Radiologist Salary Estimation**

The average annual salary for consultant radiologists in the NHS Breast Screening Programme was established using a method that reflects both the experience profile of the workforce and the latest national pay rates. To determine how experience is distributed across radiologists, we used the published audit of the NHS Breast Screening Programme (Chen et al., 2023), which reported the proportion of consultant radiologists in each experience band: approximately 11.6% have five years of experience or less, 29.9% have between five and ten years, and 58.5% have more than ten years. We then mapped each experience category to a respective pay point, ensuring our estimate reflected the real composition of the NHS breast screening workforce. The pay points for consultants are set by the NHS by years of experience as a consultant, and we chose the pay points, detailed in the NHS Pay and Conditions Circular (NHS Employers, 2023), for three years’ experience (£99,532), seven years’ experience (£105,390), and fourteen years (£119,323). This produced a national average consultant salary of £112,861 per year, representative of the typical NHS breast screening radiologist in England.

**Locum Uplifts (Adjustment for Temporary Staffing)**

NHS breast screening services depend on a blend of permanent and temporary (locum) staff to maintain service provision amid workforce shortages which are particularly large in screening services (The Royal College of Radiologists, 2023). To reflect this in our cost model, we adjust staff costs upwards to account for the higher pay rates associated with locum coverage. The key parameters for this adjustment are the proportion of each staff group working as locums (locum share) and the pay differential between locum and substantive staff.

For consultant radiologists, recent data from the Royal College of Radiologists Clinical Radiology Census Report 2023 (The Royal College of Radiologists, 2023) confirm that locums now make up 10% of the total consultant workforce in clinical radiology. Notably, this locum rate matches the vacancy rate for consultant radiologist posts, demonstrating that unfilled permanent posts are typically covered by locum staff. This pattern is intuitive: when NHS trusts are unable to fill substantive positions, they frequently rely on locum staff to ensure that clinical services are maintained. As a result, the proportion of locum staff closely mirrors the underlying vacancy rate, since almost every vacancy is temporarily backfilled given the high demand for screening services.

For radiographers, there is no direct published evidence regarding the specific locum rate in breast screening. However, that service managers respond to workforce gaps in similar way as with radiologists, we assume the locum rate for Band 7 radiographers is equivalent to their vacancy rate, which stands at 9% (Society of Radiographers, 2021).

The pay differential for locum staff is estimated using published NHS and BMA pay scales. Locums are typically paid an hourly rate or weekly rate for their services, which often exceeds substantive staff rates. For radiographers, this differential is calculated as the difference between the starting and highest pay points within Band 7, using a weighted average to reflect the workforce distribution across London and non-London settings, and the corresponding salary uplift of those working in London, using the approach outlined earlier. The regionally weighted top of the Band 7 pay scale is £26.20 per hour and the bottom is £22.97 per hour, which gives the locum pay differential of £3.23 for radiographers, or a 14.06% uplift to salary.

For consultant radiologists, the locum pay premium is derived from BMA and NHS salary guidance. The 2023 BMA Pay and Conditions Circular specifies a starting specialist doctor salary of £46,958 annually (about £900.56 per week), while the locum rate is £1,007.50 per week (BMA, 2023), which is a 11.87% uplift. We then calculate an uplift factor to represent the increased cost resulting from the use of locum staff within each profession. For radiologists, this involves applying a 10% locum share with a pay premium of 11.87% above the substantive rate, which translates to an average increase in the cost per read of approximately 1.19%. For radiographers, using a 9% locum share and a pay premium of 14.06%, the average cost per read increases by about 1.27%. These adjustment factors for each staff group are then applied to the respective annual staffing costs of each staff type prior to calculating the annual salary cost of ‘composite reader’. This explicit adjustment ensures our cost model realistically accounts for staff shortages and reliance on higher-cost locum coverage, which is a documented and growing feature of UK radiology staffing. Even though the percentage increases seem small (~1.2%), they can meaningfully impact total program costs given the large volume of screenings (millions of reads per year).

Using the most recent salary and workload data (Chen et al., 2023), we calculate the cost per individual mammogram read by dividing the locum-adjusted salaries by the average annual number of reads per staff member. To reflect the real-world mix of professionals contributing to the breast screening service, we construct a composite reader that blends the costs of radiologists and radiographers in proportion to their observed share of the total reading workload 54.4% for radiologists and 45.6% for radiographers, based on the most recent national audit. By weighting each group’s cost per read accordingly, we derive a composite cost per read of £25.11. We apply this £25.11 figure as the representative cost of one reading event in the current system. This figure will be important when we calculate the cost per screen under different AI pathways replacing human screen readers.

**Cost per Screen in the Current NHS Breast Screening Programme (Double Human Reading)**

Disagreement (Arbitration) Rate

Under the current NHSBSP protocol, two independent readers examine every screening mammogram. If their assessments (normal vs. recall decision) conflict, an arbitration step by a senior reader (usually a consultant radiologist) is required to make the final decision. Taylor-Phillips et al. (2016) reported the empirically observed disagreement frequencies in the NHSBSP: overall, 3.48% of cases require arbitration (Taylor-Phillips et al., 2016). This means approximately 1 in 29 double-read mammograms will consume a third read by a consultant for discrepancy resolution. We account for the cost of this third read by adding 3.48% of the composite reader cost to the overall per-screen cost, reflecting the proportion of cases requiring arbitration. By using a real-world derived rate, we ensure our model’s cost per screen accounts for the extra workload generated by double reading’s occasional disagreements. We assume that in the AI-only reading scenario (where no human readers are involved) there is no possibility of reader disagreement, and therefore the additional arbitration cost is not applied. However, false positives may still occur in this pathway and are accounted for through the modelled specificity of screen accuracy using AI.

**Calculation of Total Staff Cost per Screen Across the Strategies**

There is no specific NHS Reference Cost code that directly corresponds to a screening mammogram. Instead, the closest available code encompasses a range of imaging techniques. The most relevant code, in terms of description, is for “Direct Access Plain Film” within a “Diagnostic Imaging Service,” which is costed at £41 per episode according to the 2022/23 National Cost Collection (Currency Code: DAPFI, “Direct Access Plain Film”). This tariff covers the direct costs of the procedure, including technologist time, consumables, equipment use, and the initial reading of the image. However, the cost of interpretation by a second radiologist or radiographer is not fully captured by this tariff. We add to this the cost per screen of an additional interpretation, specifically the second routine read and any arbitration, as an extra staff cost.

For comparison across alternative screening pathways, staff cost is always calculated incrementally against the baseline of the one read included in the tariff. This costing method ensures that each model is evaluated consistently and that there is no double counting of the staff time or tariff elements. Arbitration costs are only included when two independent human readings are present. Where only one or no human read is performed, arbitration does not occur. The following calculations refer to mammography costs only, other screening costs such as AI licensing, AI integration, and further assessment diagnostic costs are considered in the model separately and vary by the intervention (mode of incorporating AI).

(1) Current NHS Double-Reading (two human readers, arbitration as needed): Every screen incurs an additional human read (over the first one covered by the tariff) and, in a small percentage, an arbitration read.

(2) AI and One Radiologist (AI as second reader, arbitration if AI and human disagree): In this model, only one human reader is required, and the cost of that read is included in the tariff. Arbitration is still needed if the AI and the radiologist disagree, and we assume the same arbitration rate (3.48%) as for two human readers.

(3) AI-Only, Fully Autonomous Reading: Here, no routine human reading is performed, the AI system alone interprets the images. In this case, the tariff’s “included” human read is not needed, so a reader cost is subtracted from the mammography screen baseline. No arbitration is needed, as there is only one read of the screen. It should be noted that fully autonomous AI reading is not currently permitted under NHS screening regulations; this scenario is modelled here as a theoretical exploration for future policy consideration.

(4) Two Radiologists and AI (AI as adjunct, both humans read every screen): Both human readers are retained, with AI used as an adjunct or triage tool. The presence of AI does not reduce human reading, so the staff cost is identical to the current system: one extra routine read and arbitration.

In summary, this analysis distinguishes between the technical cost of conducting a mammogram, as reflected in NHS tariff data, and the separate labour cost of image interpretation. For all pathway comparisons, costs are calculated relative to the one-read baseline embedded within the tariff, ensuring that routine interpretation costs are not double-counted.

To our knowledge, this is the first economic evaluation to micro-cost mammography image interpretation by establishing staff read time, and the first to cost staff read time using workforce involvement in breast screening in the UK. Specifically, we have accounted for actual workforce composition, considering job mix (radiologist or radiographer), experience, location, and the use of locum staff, thereby producing the most realistic estimate to date. The use of empirical rates (e.g., 3.48% disagreement) and clearly sourced cost inputs ensures the model is fully transparent, allowing for scrutiny or adaptation to other healthcare settings.

By applying the same cost per read across pathways, the model ensures a fair comparison. We are essentially asking: If the NHSBSP transitions some of the reading work to AI, how much salary cost is avoided, and how much new cost is added*?* We’ve answered the salary part above. With the current system’s per-screen cost established, we now detail the additional costs introduced by integrating AI into the screening pathway. These costs are determined on a per-screen basis and, unlike the cost of a mammogram screen, the cost does not vary by intervention scenario. That is, the below AI costs are applied uniformly to the cost of a screen in every scenario involving AI.

**AI Licensing Cost**

Per-screen licensing fees are paid to the AI software vendor for using their algorithm on each mammogram. The 2021 NICE Medtech Innovation Briefing (MIB242) collected pricing for leading AI systems in mammography (NICE, 2021). It reports that Transpara (ScreenPoint Medical), the most widely evaluated AI in NHS settings, has usage-dependent pricing that averages about £2.02 per screen after costs were uplifted from the reported 2020 /2021 prices to 2022/23 values using the Hospital and Community Health Services inflation index (PSSRU, 2023). In our base scenario, we use £2.02 as the per-screen AI license cost for Transpara, as it is based in the actual negotiated operating contract rates with the NHS.

**AI Integration, Governance and Maintenance Costs**

For our primary analysis, we focus on Transpara, using a per-screen cost of £2.02. This choice is based on a National Institute for Health and Care Excellence (NICE) report that finds that, as of 2023, four NHS trusts had adopted Transpara, indicating real-world uptake within the NHS. Additionally, among the three AI software solutions considered in the NICE report, Transpara has the highest per-screen cost. Using this higher cost represents a conservative modelling assumption, which is advantageous in health economic analysis because it reduces the risk of overestimating cost-effectiveness.

Integrating an AI system into NHS infrastructure incurs nontrivial costs related to IT expenses required for the AI system and governance and compliance costs, which is the recurring NHS overhead associated with data protection, regulatory approvals, validation of the AI for its intended purpose, and ongoing monitoring. To our knowledge, this is the first study in the literature to explicitly introduce these costs into an economic analysis of breast screening, offering a more realistic assessment of AI adoption in breast screening.

Our model takes this expense from a recent trial, the EMRAD AI in Breast Screening Project (2020), which piloted AI integration in two NHS screening sites, and uplift the costs from 2019/2020 prices to 2022/23 using the Hospital and Community Health Services inflation index (PSSRU, 2023). The report for the EMRAD trial presents these expenses in broad categories, we have extracted and specified costs wherever possible based on the available descriptions.

An external partner (Faculty AI) to the NHS sites provided a secure machine learning environment at £23,079 (2022/23 prices) for two sites. This was the related to establishing and continually monitoring a secure machine learning environment for handling large data volumes. Scaling that to all 76 screening gives an estimated £876,990 national cost, which amount to a mean per-screen cost of £0.43 based on 2,031,091 annual screens in England (NHS, 2024). Additional costs related to further developing and testing the AI model, and ongoing support of its deployment in clinical practice including integrating the AI with medical imaging were reported in the EMRAD trial from the vendor GE Healthcare. When scaled to 76 units and expressed as a cost per screen amounts to £1.64. Finally, the reported overhead and labour costs related to IT management equated to £1.31 (overhead) and £0.50 (labour) when expressed per screen. After some years of AI integration to the NHS is plausible these costs would fall, however we adopt a conservative assumption that these costs do not drop in subsequent years, and test in sensitivity analysis a doubling and halving of these costs. Together, the total environment running costs of integrating AI amounts to £3.89 per screen. When adding in the licensing costs (£2.02 per screen), total AI cost per screen is £5.91. After some years of AI integration to the NHS is plausible these costs would fall, however we adopt a conservative assumption that these costs do not drop in subsequent years.

Before AI implementation, we assume a core group of staff are involved in decision-making to agree on workflow adjustments, such as reallocating staff roles in response to AI-assisted reading replacing human readers at the site. For each screening unit, we assume this requires 2 hours each from one consultant radiologist, one advanced radiographer, and two IT or administrative staff. The combined hourly costs for these staff across all 76 screening units are divided by the national annual screening volume (2,031,091 screens) resulting in an estimated increase of £0.03 per screen in the first year. This calculation applies the previously discussed reader salary rates and costs one administrative staff member at NHS Band 5 (£16.60 per hour) and another at Band 6 (£20.53 per hour), using the regional adjustment outlined earlier of 76.9% of the screen workforce located outside London.

The costs outlined in this appendix represent the incremental direct cost to the NHS for implementing AI in screening. It does not include any downstream savings or costs (for example, if AI causes more or fewer recalls, biopsies, cancers detected and so forth). These are accounted using the economic model, in a full economic evaluation.

**Estimation of Cancer Treatment Costs**

The decision model distinguishes costs by histological subtype (DCIS vs. invasive breast cancer), by stage (1–4), by age group (< 65 vs. ≥ 65), and by year since diagnosis (1–9). All costs are presented in 2022/23 GBP, inflated from their original reported years using the Hospital & Community Health Services (HCHS) inflation index. Only direct medical costs for survivors are included, hospital costs at end of life are applied separately. Table A13 is a summary table of all non-treatment healthcare resource costs used in the model, presented in 2022/23 GBP and detailing when each cost is applied, the relevant NHS tariff codes or sources, and the associated unit values.

Table A13. Summary Table of All Non-Treatment Costs

| Resource Component | How/When Applied in Model | 2022/23 Tariff Code/Source | Value (£) | Notes/Clarification |
| --- | --- | --- | --- | --- |
| Screening invitation | All eligible, screened women, each round | NHS Health Check, inflation adjusted (Gidlow et al., 2019) | 0.731 | Uplifted from 2017/18, see Appendix |
| Mammogram | Every screen, regardless of outcome | DAPFI, 812 (NHS NCC) | 41 | Image acquisition only, staffing detailed elsewhere |
| Ultrasound | As indicated in further assessment after screen | RD41Z (NHS NCC) | 68 | Per procedure, used in further assessment |
| Core biopsy | After assessment, age- and outcome-specific | YJ04Z/YJ14Z/YJ13Z/YJ09Z (weighted mean) + pathology | 373 | Weighted average, includes £195 pathology cost |
| Open biopsy | After assessment, age- and outcome-specific | Service Code 103 (Breast Surgery) + pathology | 400 | Breast Surgery Service tariff plus £195 pathology |
| Biopsy sample processing | Added to all biopsies unless already included | CMDTB, Medical Oncology Service (NHS NCC) | 195 | Included in core/open biopsy totals above |
| MRI | True positives, as part of triple assessment | RD02A (IMAGCDC) (NHS NCC) | 391.63 | Per procedure |
| GP consultation | All symptomatic detected cancers | Unit Costs of Health and Social Care (Jones et al., 2024) | 49 | Based on mean 9.22 min consult, 2023 PSSRU |
| MDT (management team) | All true positive cases | CMDTB, Medical Oncology Service (NHS NCC) | 110 | Multidisciplinary team meeting |

NHS NCC: NHS National Cost Collection

**First-Year Treatment Costs**

First-year costs for invasive breast cancer (Stages 1–4) were taken from Wills et al. (2024), who report mean per-patient costs of surgery, radiotherapy, and systemic therapy for UK patients diagnosed between 2016 and 2018. These prices and uplifted to 2022/23 GBP using the Hospital and Community Health Services (HCHS) inflation index (PSSRU, 2023). DCIS costs were set at 40 percent of the Stage 1 cost, based on Canadian activity-based costing data from Wilkinson et al. (2023). The first-year costs are summarised in Table A14.

Table A14. First-Year Treatment Costs (2022/23 GBP)

| **Cancer Type / Age** | **Cost (£)** |
| --- | --- |
| DCIS < 65 | 3,963 |
| Stage 1 < 65 | 9,908 |
| Stage 2 < 65 | 14,445 |
| Stage 3 < 65 | 18,546 |
| Stage 4 < 65 | 30,026 |
| DCIS ≥ 65 | 3,963 |
| Stage 1 ≥ 65 | 9,908 |
| Stage 2 ≥ 65 | 14,445 |
| Stage 3 ≥ 65 | 18,546 |
| Stage 4 ≥ 65 | 30,026 |

**Follow-Up Costs (Years 2–9)**

Follow-up costs were established using a proportions-based approach, whereby annual costs were expressed as proportions of first-year costs and then applied by stage and age group accordingly. The proportions were based on annual cost data for years following diagnosis for Stages 1 and 2, and Stages 3 and 4 from Laudicella et al. (2016). This data is based on patient level costs in England for breast cancer diagnoses between 2001 and 2010. They report mean follow-up costs by year (1–9), stage group, and age group, but does not include palliative care costs which we include separately. To establish the proportions relative to first-year costs:

1. The combined Stage 1&2 first-year cost was defined as the average of the Stage 1 and Stage 2 first-year costs from Table 1; similarly for combined Stage 3&4.
2. Each year’s combined follow-up cost (Laudicella et al. 2016) was divided by the corresponding combined first-year cost to establish a year-specific proportion.
3. These proportions were applied to the stage specific first year costs we have taken from Wills et al. (2024) and Wilkinson et al. (2023). Laudicella et al. report follow-up costs only for combined stage groups (Stages 1&2 and Stages 3&4), therefore the resulting proportions were applied uniformly to the stage-specific first-year costs derived from Wills et al. (2024) and Wilkinson et al. (2023), treating Stage 1 and Stage 2 (and likewise Stage 3 and Stage 4) equivalently.

This method both preserves the relative decline in follow-up costs observed empirically and ensures ordering of costs (higher stage and older age yield higher costs). The proportion of first year treatment cost incurred in each follow up year are shown in Table A14 (Data source: Laudicella et al. 2016).

Table A15. Proportion of first year treatment cost incurred in each follow up year

| Year after Diagnosis | Stages 1–2 < 65 | Stages 1–2 ≥ 65 | Stages 3–4 < 65 | Stages 3–4 ≥ 65 |
| --- | --- | --- | --- | --- |
| 2 | 0.312 | 0.333 | 0.434 | 0.415 |
| 3 | 0.182 | 0.284 | 0.284 | 0.36 |
| 4 | 0.151 | 0.294 | 0.22 | 0.332 |
| 5 | 0.15 | 0.273 | 0.213 | 0.336 |
| 6 | 0.144 | 0.286 | 0.199 | 0.316 |
| 7 | 0.13 | 0.272 | 0.197 | 0.33 |
| 8 | 0.128 | 0.281 | 0.192 | 0.279 |
| 9 | 0.119 | 0.29 | 0.139 | 0.333 |

Applying the proportions from Table A15 to the first-year values in Table A14 produces the annual cost matrix for years 1–9 (in 2022/23 GBP) used in the model (Table A16).

Table A16. Annual Treatment Costs by Stage, Age, and Year After Diagnosis

| **Year** | **DCIS < 65** | **S1 < 65** | **S2 < 65** | **S3 < 65** | **S4 < 65** | **DCIS ≥ 65** | **S1 ≥ 65** | **S2 ≥ 65** | **S3 ≥ 65** | **S4 ≥ 65** |
| --- | --- | --- | --- | --- | --- | --- | --- | --- | --- | --- |
| 1 | 3,963 | 9,908 | 14,445 | 18,546 | 30,026 | 3,963 | 9,908 | 14,445 | 18,546 | 30,026 |
| 2 | 1,238 | 3,095 | 4,513 | 8,058 | 13,045 | 1,319 | 3,298 | 4,809 | 7,689 | 12,448 |
| 3 | 720 | 1,801 | 2,625 | 5,268 | 8,528 | 1,125 | 2,812 | 4,099 | 6,678 | 10,811 |
| 4 | 600 | 1,500 | 2,187 | 4,084 | 6,612 | 1,163 | 2,908 | 4,240 | 6,159 | 9,972 |
| 5 | 596 | 1,491 | 2,174 | 3,957 | 6,406 | 1,084 | 2,709 | 3,949 | 6,229 | 10,085 |
| 6 | 571 | 1,426 | 2,080 | 3,684 | 5,965 | 1,134 | 2,835 | 4,134 | 5,862 | 9,491 |
| 7 | 514 | 1,285 | 1,874 | 3,646 | 5,904 | 1,076 | 2,691 | 3,923 | 6,115 | 9,901 |
| 8 | 507 | 1,269 | 1,850 | 3,564 | 5,771 | 1,113 | 2,783 | 4,058 | 5,169 | 8,369 |
| 9 | 472 | 1,179 | 1,719 | 2,574 | 4,167 | 1,150 | 2,875 | 4,191 | 6,176 | 9,999 |

**Hospital Costs at End of Life**

Hospital costs at end of life are applied in the model whenever an individual dies, either from breast cancer or from other natural causes, the costs applied distinguishing between breast cancer non-survivors and decedents from other causes.

Hospital and community palliative care costs were sourced from Diernberger et al. (2023), who analysed Scottish inpatient and outpatient data for all decedents aged 60 and above between January 2011 and December 2017. The study reports mean end-of-life costs for deaths attributed to breast cancer and for deaths from other causes. These figures were averaged across the seven years of data, then inflated from their original 2018/19 price base to 2022/23 GBP using the HCHS index. A summary of the costs are provided in Table A17.

Table A17. Palliative Care Hospital Costs by Age Band and Cause of Death

| Age Band | Breast Cancer Death (£) | Other-Cause Death (£) |
| --- | --- | --- |
| 60–64 | 16,979 | 14,734 |
| 65–69 | 17,870 | 14,346 |
| 70–74 | 13,994 | 12,860 |
| 75–79 | 12,016 | 12,720 |
| 80–84 | 10,208 | 11,829 |
| 85–89 | 7,901 | 10,609 |
| ≥ 90 | 7,091 | 9,878 |

For breast cancer non-survivors in the death year, the age-specific hospital costs are compared with cancer treatment cost in that year, and the higher of the two is used. If death is from another cause, the age-specific non-cancer palliative cost is used. This approach ensures that the model captures the full hospital-based resource use at the end of life, reflecting higher intensity of care in cancer deaths and accounting for age-related cost variation.

**Estimation and Application of Screening-Related Costs**

This section provides a detailed account of all screening and diagnostic costs incorporated into the breast cancer screening model. Costs reflect 2022/23 GBP values.

**Screening Invitation**

All women who are eligible for the screening programme are assigned the cost of a screening invitation, regardless of diagnostic outcome. Those who attend a screening appointment receive the full cost of a mammogram, which is assigned for every screen regardless of subsequent recall, assessment, or diagnosis.

The per-person cost for issuing a screening invitation, as used in this model, is taken from cost data reported in Gidlow et al. (2019). In that study, costs were calculated based on observed resource use across nine general practices participating in a randomised controlled trial of invitation methods for NHS Health Checks, with the base year for reported costs being 2017/18. To ensure comparability with all other cost inputs in the model, the screening invitation cost was uplifted to 2022/23 values using the Hospital and Community Health Services inflation index (PSSRU, 2023), with the resulting value of £0.73 per screening invitation.

**Diagnosis Unit Costs**

All true positive cases are assigned the cost of a breast cancer management team meeting at £110 (Code: CMDTB, Medical Oncology Service) from NHS procedure unit costs (NHS England, 2024). All women presenting with symptomatically detected breast cancer, as opposed to cancer detected at a screening appointment, are assigned the cost of a standard GP consultation (£49 per consultation, based on a mean duration of 9.22 minutes), drawn from the 2023 Unit Costs of Health and Social Care manual (PSSRU, 2023). These costs are summarised in Table A18.

**Further Assessment Following a Positive Screen**

Among women with a positive screening result (including true positives at a screening appointment, false positives at a screening appointment, and symptomatic presentations), further assessment costs are assigned probabilistically from three imaging modalities: a second mammography, breast ultrasound and breast magnetic resonance imaging (MRI). These are the further assessment imaging procedures recommended in the UK national screening programme.

There is a lack of available data from UK sources regarding the rates of further assessment imaging procedures following a mammography screen. Consequently, the probabilities of undergoing additional diagnostic imaging after are taken from a US-based study which analyses utilisation patterns of breast imaging and diagnostic procedures following screening mammography (Vlahiotis et al., 2018). There is a probability of 0.329 for a second mammography, 0.582 for ultrasound, and 0.014 for MRI which together account for 0.925 of cases. The remaining women are assumed to receive only a clinical examination.

Ultrasound and MRI costs were calculated by multiplying the probabilities of use by the corresponding 2022/23 national average NHS procedure unit costs (NHS England, 2022). The NHS National Cost Collection provides standardised, annually reported data on the average costs of delivering healthcare services across NHS providers in England, and is commonly used in health economic analyses to inform cost-effectiveness evaluations by offering a consistent and nationally representative basis for estimating healthcare resource use and costs. The cost of mammography was estimated using a micro-costing approach that accounts for both image acquisition and staff time required for image interpretation detailed above. The reading time component depends on the use of AI and therefore varies depending on the intervention. The cost for image acquisition is based on tariff code DAPFI, 812, which reflects the NHS rate for ‘Direct Access Plain Film’ within a ‘Diagnostic Imaging Service’ with a cost of £41. Screening related costs and their sources are summarised in table A18

Table A18. Summary of screening related costs

| **Screen related resource** | **2022/23 Tariff Code** | **Unit Cost (£)** | **Reference** |
| --- | --- | --- | --- |
| GP consultation | Unit Costs of Health and Social Care | 49 | Jones et al., 2024 |
| Screening invitation | NHS Health Check, inflation adjusted | 0.731 | Jones et al., 2024 |
| MDT, TP only | CMDTB, Medical Oncology Service | 110 | NHS NCC 2022/23 |
| Mammography | DAPFI, 812 | imagine acquisition is £41 | NHS National Cost Collection 2022/23 |
| Ultrasound | RD41Z | £68 | NHS National Cost Collection 2022/23 |
| MRI | RD02A (IMAGCDC) | £391.63 | NHS National Cost Collection 2022/23 |

**Biopsy and Pathology**

Following further assessment, women may undergo core needle biopsy or open surgical biopsy, with probabilities dependent on age and screening outcome (true positive, false positive). The age specific probabilities for core and open biopsy rates following a false positive mammogram screening were taken from (Shen et al., 2017). The age specific probabilities for core and open biopsy rates following a true positive mammogram are based on 2022/23 NHS breast screening programme audit data (NHS Digital, 2024). The probabilities, summarised in Table A19, were calculated by dividing the number of women referred for further assessment in the National Breast Cancer Screening Programme by the total number of biopsies performed.

Table A19. Probability of Biopsy by Age Group and Screening Outcome

| **Age Band** | **Core Biopsy (TP)** | **Open Biopsy (TP)** | **Core Biopsy (FP)** | **Open Biopsy (FP)** |
| --- | --- | --- | --- | --- |
| 50–52 | 0.483 | 0.013 | 0.014 | 0.078 |
| 53–54 | 0.444 | 0.011 | 0.011 | 0.089 |
| 55–59 | 0.48 | 0.012 | 0.01 | 0.09 |
| 60–64 | 0.514 | 0.009 | 0.011 | 0.089 |
| 65–70 | 0.546 | 0.011 | 0.01 | 0.09 |
| 70+ | 0.595 | 0.011 | 0.01 | 0.09 |

The probabilities in Table A19, which depend on age of a positive detection, were then applied to estimate whether an individual would receive a core or open biopsy. The resulting procedure was multiplied by the corresponding 2022/23 national average NHS procedure unit costs (NHS England, 2024) to calculate the associated cost.

**Biopsy Unit Costs**

The cost of core needle biopsy was calculated by weighting the unit costs of the individual biopsy types by their respective volumes (number of examinations) reported in the 2022/23 NHS Reference Costs (Currency Codes: YJ04Z, YJ14Z, YJ13Z, YJ09Z). This weighted average approach ensures that biopsy types with higher utilisation contribute proportionally more to the overall average cost, thereby providing a more accurate reflection of the true average expenditure per biopsy. These costs are summarised in Table A20.

Table A20. Summary of biopsy costs.

| Procedure Description | Currency Code | No. Exams | Unit Cost (£) |
| --- | --- | --- | --- |
| Core Needle Biopsy of Axillary Lymph Nodes | YJ04Z | 60 | £104 |
| Stereotactic Core Needle Biopsy of Lesion of Breast | YJ14Z | 21 | £447 |
| Ultrasound Guided Core Needle Biopsy of Lesion | YJ13Z | 234 | £97 |
| Vacuum Assisted Biopsy or Excision of Lesion | YJ09Z | 812 | £347 |

For open biopsy, since specific NHS reference cost data were not available in the dataset, the model uses the national average outpatient breast surgery service tariff of £205 (Service Code 103 - Breast Surgery Service, Consultant Led). To capture the full cost associated with biopsy procedures, an additional £195 is added to each biopsy cost to reflect the mean expense of pathology sample processing and consultant review. This is the unit cost reported for the Medical Oncology Service (Service Code 370, Consultant Led, Department Description: Medical Oncology Service) in 2022/23 national average NHS procedure unit costs (NHS England, 2024). After including this additional cost, the costs applied for core and open biopsy are £373 and £400. The cost of mammography was estimated using a micro-costing approach that accounts for both image acquisition and staff time required for image interpretation. The reading time component varies depending on the intervention, particularly the use of AI, as modelled. The base cost for image acquisition is aligned with tariff code DAPFI, 812, which reflects the NHS rate for ‘Direct Access Plain Film’ within a ‘Diagnostic Imaging Service’ at a Unit cost of £41.

**Health Utility**

In this appendix section, we provide a detailed account of all utility inputs incorporated into our breast cancer screening cost–utility model, including the underlying evidence and methodological considerations. In each model cycle, individual QALY accrual combines the age-specific baseline utility, the first-year treatment decrement (if within year 1 post diagnosis), the year-specific long-term decrement for ongoing cancer survivorship, an end-of-life decrement and any false positive screening-related decrement in screening years.

EQ‑5D‑5L utility values for women in the general population of England were used to represent health utility without diagnosed cancer, and were obtained from McNamara (2023), and are summarised in Table A21. These reference utilities were stratified by five-year age bands and form the basis to which all cancer and screening related utility decrements were applied in each annual cycle.

Table A21. General Population Utilities (McNamara, 2023).

| **Age Group** | **EQ‑5D‑5L Utility** |
| --- | --- |
| 45–49 | 0.806 |
| 50–54 | 0.798 |
| 55–59 | 0.791 |
| 60–64 | 0.776 |
| 65–69 | 0.777 |
| 70–74 | 0.773 |
| 75–79 | 0.726 |
| 80–84 | 0.701 |
| ≥ 85 | 0.666 |

For ductal carcinoma in situ (DCIS) and stage 1 breast cancer, short‑term (first‑year) utility decrements were derived by integrating Australian patient‑elicited EQ‑5D‑5L treatment utilities (Bromley et al., 2019) with UK general population norms and English treatment distributions. Bromley et al. reported mean utility values for women aged 66.2 years with screen‑detected DCIS or early invasive breast cancer: 0.767 for breast conserving surgery alone, 0.606 for mastectomy, and 0.699 for breast conserving surgery plus radiotherapy. These values were converted into decrements by subtracting from the age‑matched reference utility (EQ‑5D‑5L = 0.776 for women in the general population aged 66.2; McNamara et al., 2023), yielding treatment decrements of 0.009, 0.170 and 0.077, respectively.

Procedure‑specific probabilities for women with cancer in England aged 50–69 was extracted from the National Audit of Breast Cancer in Older Patients 2022 report (NABCOP, 2022). For DCIS disease, 22% undergo breast conserving surgery alone, 60% receive radiotherapy, and 18% undergo mastectomy. These decrement weighted probabilities summarised in Table A22.

For stage 1 invasive disease, distributions were from English women in 2014 to 2015 (Sun et al. 2020): 70% breast conserving surgery plus radiotherapy, 16% mastectomy, and 9% surgery with chemotherapy with the remaining women (5%) assumed to have breast conserving surgery alone. The utility impact of chemotherapy beyond surgery was calculated by combining an age‑adjusted chemotherapy decrement (0.205; Wang et al., 2022) with the surgery‑alone decrement (0.009), yielding 0.214 for chemotherapy in addition to surgery. Multiplying each treatment‑specific decrement by its corresponding probability and summing the health utility produced an overall first‑year utility decrement of 0.0788 for DCIS and 0.1008 for stage 1. To eestablish the utility decrement in the first year for stages 2, 3 and 4, we applied the increase in the in EQ-5D-5L utility score decrement, relative to that of stage 1, for women with breast cancer (Moshina., 2022): an increase of the decrement of 0.03 (for stage 2), 0.04 (stage 3) and 0.06 (stage 4).

The utility decrement change by time since diagnosis were informed by a randomised controlled trial that reported EQ‑5D‑3L quality-of-life differences of women age 50 and above at 1, 2, 3, 5 and 10 years post diagnosis compared to age matched general female population (Roine, 2021). In that study, mean declines in health‑related quality of life for women aged ≤50 and >50 were statistically significant over time (age p=0.027; time p=0.053; age multiplied by time interaction p<0.001). We derived proportional change factors at each follow-up interval by expressing the mean difference in health utility relative to the first-year value. These factors are 1.00 in year 1, 0.94 in years 2 and 3, 0.76 in year 5, and 1.12 in year 10. We used a linear interpolation, based on the difference in the factors between years 3 and 5, and again between years 5 and 10, to arrive a factor for every year until year 10. The factors were applied to the first-year utility decrements for DCIS and stage 1 to model health utility over ten years. The resulting decrements from female general population health utility by stage and duration are shown in Table A22.

The ten-year decrement was extended to subsequent years until death. An end-of-life utility decrement of 0.026 is applied during the final six months of life, which is the incremental difference in EQ-5D-3L health utility decrements between breast cancer patients with metastatic disease and those receiving palliative care, as reported by Rautalin et al. (2018).

Table A22. Utility Decrements by Stage and Year Since Diagnosis

| **Year** | **DCIS** | **Stage 1** | **Stage 2** | **Stage 3** | **Stage 4** |
| --- | --- | --- | --- | --- | --- |
| 1 | 0.0788 | 0.1008 | 0.1308 | 0.1408 | 0.1608 |
| 2 | 0.0741 | 0.0949 | 0.1231 | 0.1325 | 0.1514 |
| 3 | 0.0741 | 0.0949 | 0.1231 | 0.1325 | 0.1514 |
| 4 | 0.0672 | 0.0860 | 0.1116 | 0.1201 | 0.1372 |
| 5 | 0.0602 | 0.0771 | 0.1000 | 0.1077 | 0.1230 |
| 6 | 0.0658 | 0.0842 | 0.1093 | 0.1176 | 0.1343 |
| 7 | 0.0714 | 0.0913 | 0.1185 | 0.1276 | 0.1457 |
| 8 | 0.0769 | 0.0984 | 0.1277 | 0.1375 | 0.1570 |
| 9 | 0.0825 | 0.1056 | 0.1370 | 0.1474 | 0.1684 |
| 10 and greater | 0.0880 | 0.1127 | 0.1462 | 0.1574 | 0.1797 |

**Health Utility Losses from False Positive Screening Results**

We assume that any health utility losses associated with true positive cancer detection are captured in the first-year cancer health utility decrements which are applied at cancer detection. For women who receive a false positive result from breast cancer screening, the model simulates subsequent follow-up procedures based on age-specific probabilities. Each woman with a false positive result is assigned to one of three possible follow-up pathways. In the first pathway, the woman receives additional imaging (e.g., repeat mammogram or ultrasound) but does not proceed to MRI or biopsy. In the second, the woman undergoes a biopsy procedure (either core biopsy or open biopsy). In the third, the woman is referred for a magnetic resonance imaging (MRI) scan.

The assignment to each pathway is determined by probabilities stratified by age band, shown in Table A23. Probabilities are based on data from the further-assessment imaging table for magnetic resonance imaging, and the sum of age-specific probabilities for core and open biopsy among false positives. The remaining proportion is assigned to additional imaging only.

Table A23. Probabilities for Follow-up Procedures After a False Positive Screening Result

| Age Band | Probability of MRI | Probability of any biopsy (core or open) | Probability of Imaging Only (neither MRI nor biopsy) |
| --- | --- | --- | --- |
| 50–59 | 0.014 | 0.09175 | 0.89425 |
| 60–64 | 0.014 | 0.1004 | 0.8856 |
| 65+ | 0.014 | 0.1 | 0.886 |

The probability of MRI is based on the percentage of women who receive an MRI as a follow up assessment after first being screened with mammography (Vlahiotis et al, 2018) . The probability of biopsy is the sum of age-specific rates for core and open biopsy rates following a false positive mammogram screening (Shen et al., 2017). The reported rates provide an evidence-based estimate of the likelihood of a biopsy following a false positive screening result. The probability of imaging only is calculated as 1 minus the sum of the MRI and biopsy probabilities. Women assigned to each pathway are modelled as experiencing the corresponding diagnostic pathway QALY losses for false-positive outcomes, according to their age group. The health utility decrement from the procedures and the assumed duration the decrement lasts (ten days), were sourced from Matza et al. (2024). These decrements, summarised in Table A24, were established in Matza et al. (2024) to specifically capture the psychological burdens of the procedures and waiting for a confirmed diagnosis following a false positive in breast screening in UK women.

Table A24. Screening-Related Utility Decrements (Matza et al., 2024)

| False Positive Follow-Up Procedure | Mean Disutility | Disutility measured in terms of QALY reduction |
| --- | --- | --- |
| Mammography false positive for breast cancer; no biopsy or MRI | 0.031 | 0.0009 |
| Mammography false positive for breast cancer led to core biopsy performed | 0.058 | 0.0016 |
| Mammography false positive for breast cancer led to MRI | 0.067 | 0.0018 |

**Summary of survival in the model**

In the model simulation, after cancer detection each individual's survival is tracked on a yearly basis. To correct for lead time this is calculated from their age of symptomatic detection. For every cancer case, the model first identifies the stage at diagnosis and whether the cancer was detected through screening or symptomatically. It then selects the relevant annual survival probabilities from the lookup tables that correspond to both the stage and mode of detection. Each year, the model uses these probabilities to determine, through a random sampling process, whether the individual survives or dies from breast cancer. Non-cancer (background) mortality is modelled separately, and if a non-cancer death occurs before a breast cancer death, it takes precedence. For individuals diagnosed with DCIS, the model does not simulate death from breast cancer unless there is progression to a higher-stage invasive disease.

**Stage Specific Survival by Detection Mode**

Published breast cancer screening models often report only overall stage-specific survival or generic hazard ratios for mode of detection. Instead of relying on generic hazard ratios or outdated non-UK estimates, this model disaggregates the most recent overall stage-specific survival rates into detection mode-specific rates using real-world proportions of screen-detected and symptomatic cases, ensuring full calibration to current NHS outcomes. UK-based analyses of contemporary outcomes are especially important for informing UK national screening policy and economic analysis.

**Stage specific survival data**

Stage-specific, age-standardised five-year net survival rates for breast cancer in women in England are derived from the most recent NHS data (NHS Digital, 2023) covering diagnoses from 2016 to 2021. Net survival is defined as the proportion of women surviving breast cancer as the underlying cause, discounting deaths from unrelated causes, so the model specifically tracks cancer-specific mortality rather than all-cause mortality. The five-year net survival for stage 1 breast cancer was 98.2%, for stage 2 it was 89.5%, for stage 3 it was 72.2% and for stage 4 it was 26.6% (NHS Digital, 2023).

Table A25. Five- and Ten-Year Net Survival by Stage in Ali et al (2011)

| Stage | 5-Year Survival (%) | 10-Year Survival (%) | Ratio (10y/5y) |
| --- | --- | --- | --- |
| Stage 1 | 99 | 97 | 0.98 |
| Stage 2 | 87 | 79 | 0.91 |
| Stage 3 | 48 | 29 | 0.6 |
| Stage 4 | 14 | 5 | 0.36 |

The five-year data is preferred over long-term survival estimates because it is the most recent available, capturing outcomes during a period of significant advancements in diagnosis and treatment. As such, it more accurately reflects current UK clinical practice and standards of breast cancer care.

To estimate longer-term (ten-year) survival, the model applies the ratio of ten-year to five-year survival by stage observed in Ali et al. (2011), a UK cohort study reporting both 5- and 10-year outcomes. For example, if five-year survival for stage 1 in Ali et al. is 99% and ten-year survival is 97%, the ratio (97/99 ≈ 0.98) is multiplied by the most recent NHS Digital five-year survival figure to obtain a calibrated ten-year value under current standards. Table A25 shows the ratio multiplies applied. This method assumes that the relationship between five- and ten-year survival in modern data is similar to that observed in the Ali et al. cohort, which is the most complete published UK source. For years 6 to 9 after diagnosis, annual survival is interpolated linearly between the five-year and ten-year rates for each stage to provide a transition at a constant rate from short-term to longer-term outcomes.

Ductal carcinoma in situ (DCIS) is treated distinctly in the model. DCIS does not confer a direct risk of breast cancer-specific mortality (Narod et al., 2015). Any breast cancer deaths among women with a history of DCIS occur only indirectly, through the development of a subsequent, higher-stage invasive cancer (i.e., through recurrence or progression that is captured in the economic model). This modelling assumption ensures that deaths are not attributed to DCIS itself, but to later invasive events, as supported by epidemiological literature (Narod et al., 2015).

**Method of Calculating Survival Rates by Detection Mode**

Within this model, the probability of surviving breast cancer is determined not only by the stage at diagnosis but also by whether the cancer was detected through screening or outside of a screening appointment (clinical detection by symptoms). UK-based empirical evidence (Ali et al., 2011) shows that, at any given stage, screen-detected breast cancers are associated with better survival outcomes, and this has also been confirmed recently in the German breast screening programme (Buschmann, 2024). This observation has been supported by data from randomized controlled trials (RCTs), where, even after adjusting for tumour size, lymph node involvement, and disease stage using a Cox proportional hazards model, the method of detection remained a statistically significant independent predictor of disease-specific survival (Shen et al., 2005). Therefore, it is important that the model assigns realistic and evidence-based survival rates to screen and symptomatically detected cases of cancer (interval cancers and cases in non-attenders). The approach we take is to use real detection proportions of screen and symptomatic cancer and calibrate to the stage survival rates to ensure that the model’s outputs are directly comparable to empirical UK survival outcomes.

For each stage, the yearly (from one to ten years) stage-specific survival estimate is treated as a weighted average of the survival rates of screen-detected and symptomatically detected cancers. The weights used are based on the proportions of screen-detected non metastatic cancer (44.16%) and screen-detected metastatic cancer (6.27%) observed in the England breast screening programme between 2014 and 2019 (NABCOP, 2022). Using these weights, we estimate detection-mode-specific survival rates, one for screen-detected and one for symptomatically detected cancers, by ensuring the weighted average of the two survival estimates matches the age-adjusted, stage-based survival by year since diagnosis. This involves solving for the pair of survival rates each year since diagnosis that satisfy the following criteria. Their weighted average, using the observed detection proportions, exactly matches the actual overall survival rate for that stage, and the screen-detected survival rate must be higher than the symptomatic rate, and survival for stage 1 screen-detected cancer is capped at 100%. For every woman simulated in the model, her years of surviving breast cancer is determined by a comparison of a random number draw to the resulting cumulative mortality estimates shown in Table A26.

Table A26. Cumulative Mortality by Detection Mode

| **Year after Detection** | **Screen Stage 1** | **Screen Stage 2** | **Screen Stage 3** | **Screen Stage 4** | **Symp Stage 1** | **Symp Stage 2** | **Symp Stage 3** | **Symp Stage 4** |
| --- | --- | --- | --- | --- | --- | --- | --- | --- |
| 1 | 0 | 0 | 2.6 | 31.8 | 1.5 | 2.4 | 5.8 | 33.1 |
| 2 | 0 | 1.3 | 9.3 | 45.7 | 1.8 | 4.5 | 12.2 | 46.8 |
| 3 | 0 | 4 | 15.1 | 57.9 | 2 | 7.1 | 17.8 | 58.8 |
| 4 | 0 | 6.6 | 20.7 | 66.5 | 2.5 | 9.6 | 23.2 | 67.1 |
| 5 | 0 | 8.9 | 26.5 | 72.9 | 3.2 | 11.8 | 28.8 | 73.4 |
| 6 | 0.4 | 10.5 | 32.3 | 76.4 | 3.6 | 13.4 | 34.5 | 76.8 |
| 7 | 0.8 | 12.2 | 38.1 | 79.9 | 4.2 | 16 | 43.5 | 82.3 |
| 8 | 1.2 | 13.9 | 43.9 | 83.4 | 4.8 | 18.4 | 51.6 | 87.2 |
| 9 | 1.6 | 15.6 | 49.8 | 86.8 | 5.1 | 19.6 | 55.9 | 89.9 |
| 10 | 2 | 17.2 | 55.6 | 90.3 | 5.2 | 19.9 | 57 | 90.5 |

For stage 4 cancer we assume all women die of cancer in year 11.

**Breast-Density Assignment and Progression**

This sub section of the appendix describes how mammographic breast density is represented, assigned and updated within the simulation. Breast density (the percentage of fibroglandular tissue visible on a mammogram) is treated as a continuous, age-dependent variable. It’s role in the model is that it effects the sensitivity and specificity of mammographic screening. The breast density evidence we use is population data obtained with Volpara automated volumetric analysis and in longitudinal studies that track density change over time.

**Empirical distributions used at model entry**

Table A27 reports the age- and cancer-specific mean, standard deviation, and observed range of digital-mammography UK women for women who eventually develop screen detected cancer and matched controls of women do not develop cancer (Damiani et al., 2023). These figures show the generally declining average density, and the diagnostic density observed among women who eventually develop screen-detected cancer. These parameters define the truncated normal distributions from which individual densities are sampled.

Table A27. Percent breast-density distributions by age band and cancer status

| Age band (y) | Mean % (no-cancer) | SD | Mean % (screen-detected cancer) | SD | Min | Max |
| --- | --- | --- | --- | --- | --- | --- |
| 45–49 | 8.77 | 6.44 | 13.49 | 7.79 | 2.86 | 30.65 |
| 50–54 | 9.24 | 5.8 | 10.72 | 6.67 | 2.51 | 28.56 |
| 55–59 | 7.45 | 4.79 | 7.81 | 5.15 | 2.38 | 28.12 |
| 60–64 | 7.35 | 4.7 | 7.45 | 4.51 | 2.31 | 31.52 |
| 65–69 | 7.74 | 5.49 | 6.68 | 3.65 | 2.23 | 32.51 |
| 70–75 | 7.46 | 4.56 | 7.01 | 4.59 | 2.52 | 21.08 |

**Initial assignment and age-band updates**

At time-zero (when women enter the model at the age of their first screening invitation, age 50) the model draws one breast-density value for every five-year age band (45–49 through 70–75) for each woman. Draws use the age-appropriate distribution in Table A27, truncated to the empirical minimum and maximum. As a woman ages she does not receive a fresh random draw; instead, when she enters a new age band the model simply adopts the pre-sampled value that corresponds to that band. This procedure preserves between individual variability while reproducing the correct cross-sectional means at every age. Whether a woman will develop cancer in her lifetime is determined at time zero. As a result, cancer status is already known at the outset, and women who never develop cancer consistently remain in the 'No-cancer' breast density trajectory group throughout the study. Women who do develop screen-detected cancer retain the same pre-sampled trajectory until the age band in which their cancer becomes screen-detectable (the cancer onset age); the breast-density value for that band is taken from the “Screen-detected-cancer” distribution in Table A27. Please note that the process for determining cancer incidence at the start of the model, as well as the assignment of cancer onset age, is described elsewhere in the appendix.

**Post-diagnosis density change**

After diagnosis the model applies fixed percentage-point decrements to breast density in each subsequent age band. This is the cross sectional mean difference between age bands for screen detected cases in Damiani et al., 2023. The size of each decrement is shown in Table Table A28. The reductions are additive, so the cumulative loss after four post-diagnosis bands is about 6.8 percentage points, after which density stabilises.

Table A28. Step-wise percent-density reductions applied after screen-detected cancer diagnosis

| **Age band after diagnosis** | **Incremental reduction** | **Cumulative reduction** |
| --- | --- | --- |
| 50–54 | 2.77 | 2.77 |
| 55–59 | 2.91 | 5.68 |
| 60–64 | 0.36 | 6.04 |
| 65–69 | 0.77 | 6.81 |
| 70–75 | 0 | 6.81 |

After each age-band update the continuous percent-density figure is categorised using the Volpara Density Grade thresholds recommended by the manufacturer. Volpara Grade A corresponds to a percent-density range of less than 4.5%. Volpara Grade B corresponds to a percent-density range from 4.5% to 7.5%. Volpara Grade C corresponds to a percent-density range from 7.5% to 15.5%. Volpara Grade D corresponds to a percent-density range of 15.5% or greater. These categorical grades feed directly into the screening sensitivity and specificity components of the model.

In summary, the model assigns every woman an age-specific breast-density trajectory rooted in real-world data of mammographic screens in UK women with and without a cancer diagnosis, and updates that trajectory deterministically as she ages or, if applicable, after the onset of breast cancer. Continuous values remain within empirically observed limits and are converted to Volpara Density Grades for downstream use in the screening diagnostic accuracy. This approach reproduces both population-level density distributions.

**Model Validation**

The model was externally validated to target data from the NHS national breast screening programme. The outcomes are reported in Table A29. The model prediction was standard care (double reading by two human readers), and one validation target is the percentage of tumours that are DCIS at the time of cancer detection (13.8%). This figure is sourced from Table A4.3 in the National Audit of Breast Cancer in Older Patients 2022 annual report. However, since this report does not provide details on the TNM stage of invasive cancers at detection, the validation is limited to distinguishing between DCIS and invasive cancer. Another validation target is the percentage of screen-detected cancers. This is calculated using the number of cancers diagnosed during screening appointments (46.2%), compared to diagnoses through all other pathways, as reported in Table A4.2 of the same annual report.

For the first validation target, the proportion of DCIS among detected tumours, the model predicted 14.9%, compared to the observed rate of 13.8%. This shows a close correspondence, with the model slightly overestimating the rate by 1.1 percentage points. The second validation target, the proportion of screen-detected cancers, was also closely matched. The model predicted a rate of 51.7%, compared to the observed value of 46.2%, again showing a modest overestimation of 5.5 percentage points. This level of agreement indicates that the model reasonably approximates real-world detection patterns within the screening programme, though it slightly overpredicts detection through screening pathways.

Overall, the validation results demonstrate that the model predictions are in close alignment with empirical data, with differences that are small enough to support confidence in the model’s applicability for simulating breast cancer detection outcomes under standard care.

Table A29. Model validation results.

| **Validation target** | **Validation target outcomes** | **Model prediction** | **Source of target outcomes** |
| --- | --- | --- | --- |
| Percentage of tumours that are DCIS upon detection | 13.80% | 14.90% | National Audit of Breast Cancer in Older Patients 2022 report. Table 3.3.1. |
| Percentage of stage detected cancers | 46.20% | 51.70% | National Audit of Breast Cancer in Older Patients 2022 report. Table 3.3.1. |

**Deterministic Model results and deterministic sensitivity analysis.**

The deterministic model results, summarised in Table A30, provide a assessment of different screening strategies based on their health outcomes (measured in QALYs), costs, and net monetary benefit (NMB) at willingness-to-pay thresholds of £20,000 and £30,000 per QALY gained.

Under standard care (double reading by two human readers), the model estimates 14.67535 QALYs and costs of £5,642 per person invited. When one of the human readers is replaced with AI (double reading: 1 human + AI), QALYs increase slightly to 14.67563, and costs decrease to £5,559. This strategy led to higher NMB per person invited: £287,954 at the £20,000 threshold and £434,710 at the £30,000 threshold

Triple reading (2 humans + AI) has estimates of 14.67525 QALYs and costs of £5,644. Although it delivers a slightly larger QALY gain than standard care (0.0012) it also has higher costs (by £2.42 per person), resulting in NMBs of £287,886 and £434,651 at the two thresholds.

The AI-only strategy shows the lowest cost (£5,471) and lowest QALYs (14.67524). However, it results in the largest NMB at £20k (£288,034) and resulted in cost saving compared to standard care by £170.31 per person invited.

Table A30. Deterministic model results

| Screening strategy | Outcomes per person invited | | Screening strategy vs. standard care | | NMB (£) at £20k and £30K | |
| --- | --- | --- | --- | --- | --- | --- |
|  | QALYs | Costs (£) | QALYs | Costs (£) | @ £20k (£) | @ £30k (£) |
| Standard care | 14.67535 | 5642 | - | - | 287,865 | 434,619 |
| Double reading  (1 human + AI) | 14.67563 | 5559 | 0.00028 | - 82.92 | 287,954 | 434,710 |
| Triple reading (2 humans + AI) | 14.67652 | 5644 | 0.00117 | 2.42 | 287,886 | 434,651 |
| Single read by AI | 14.67524 | 5471 | - 0.0001 | - 170.69 | 288,034 | 434,786 |

Deterministic sensitivity analysis explores how escalating AI costs per screen influence the economic outcomes of two AI-integrated breast cancer screening strategies. The results are reported in Table A31.

As AI costs are incrementally increased by 5% up to 100%, the strategies see modest rises in total costs per person. For example, with a 5% AI cost increase, the 1 human + AI strategy cost rises to £5,560, with the NMB declining marginally to £287,953 (at £20k) and £434,709 (at £30k). A 100% increase raises the cost to £5,580 and reduces the NMB to £287,932 and £434,689, respectively. A similar pattern is observed for the 2 humans with AI strategy. At a 100% AI cost increase, the cost per person rises to £5,666, and the NMB falls to £287,865 (at £20k) and £434,630 (at £30k). Regarding the AI only strategy. At a 100% AI cost increase, the cost per person rises to £5,492, and the NMB falls to £288,013 (at £20k) and £434,765 (at £30k), representing a modest decrease of only £21 in NMB compared to the base case. Overall, this analysis demonstrates that the strategy of a single read using AI maintains its position of having the highest NMB even under substantial AI cost increases.

Table A31. Deterministic sensitivity analysis increasing the AI cost per screen.

| Proportional increase in AI costs | Screening strategy | Costs (£) per person invited to screening | NMB (£) at £20k per person invited to screening | NMB (£) at £20k per person invited to screening |
| --- | --- | --- | --- | --- |
| Base case (no change) | Double reading  (1 human + AI) | 5559 | 287,954 | 434,710 |
|  | Triple reading (2 humans + AI) | 5644 | 287,886 | 434,651 |
|  | Single read (AI only) | 5471 | 288,034 | 434,786 |
| AI costs increased by 5% | 1 human + AI | 5560 | 287,953 | 434,709 |
|  | 2 humans + AI | 5645 | 287,885 | 434,650 |
|  | AI only | 5472 | 288,033 | 434,785 |
| AI costs increased by 10% | 1 human + AI | 5561 | 287,951 | 434,708 |
|  | 2 humans + AI | 5646 | 287,884 | 434,649 |
|  | AI only | 5473 | 288,032 | 434,784 |
| AI costs increased by 15% | 1 human + AI | 5562 | 287,950 | 434,707 |
|  | 2 humans + AI | 5648 | 287,883 | 434,648 |
|  | AI only | 5474 | 288,031 | 434,783 |
| AI costs increased by 20% | 1 human + AI | 5563 | 287,949 | 434,706 |
|  | 2 humans + AI | 5649 | 287,882 | 434,647 |
|  | AI only | 5476 | 288,029 | 434,782 |
| AI costs increased by 25% | 1 human + AI | 5564 | 287,948 | 434,705 |
|  | 2 humans + AI | 5650 | 287,881 | 434,646 |
|  | AI only | 5477 | 288,028 | 434,781 |
| AI costs increased by 30% | 1 human + AI | 5565 | 287,947 | 434,704 |
|  | 2 humans + AI | 5651 | 287,880 | 434,645 |
|  | AI only | 5478 | 288,027 | 434,780 |
| AI costs increased by 50% | 1 human + AI | 5570 | 287,943 | 434,699 |
|  | 2 humans + AI | 5655 | 287,875 | 434,641 |
|  | AI only | 5482 | 288,023 | 434,776 |
| AI costs increased by 75% | 1 human + AI | 5575 | 287,938 | 434,694 |
|  | 2 humans + AI | 5660 | 287,870 | 434,635 |
|  | AI only | 5487 | 288,018 | 434,770 |
| AI costs increased by 100% | 1 human + AI | 5580 | 287,932 | 434,689 |
|  | 2 humans + AI | 5666 | 287,865 | 434,630 |
|  | AI only | 5492 | 288,013 | 434,765 |

**Probabilistic Results with Single reading by AI strategy excluded.**

The incremental QALYs and incremental Costs of each AI based screening strategy compared to usual care is presented in Figures A1 to A3. The probability that each screening strategy is most cost-effective, compared to each other and across a range of willingness-to-pay (WTP) thresholds per QALY gained is presented in Table A32. This is illustrated in a Cost-effectivness acceptable curve in the main paper.

Figure A1. Scatter plot of the incremental QALYs and costs of double reading by one human and AI compared to standard practice (double reading by two humans)

Figure A2. Scatter plot of the incremental QALYs and costs of a single read by an AI compared to standard practice (double reading by two humans)

Figure A3. Scatter plot of the incremental QALYs and costs of triple reading (two humans and AI) vs. standard practice (double reading by two humans)

Table A32 shows that, at the typical UK cost-effectiveness threshold of £20,000, Single Reading by AI is overwhelmingly favoured, being most cost-effective in all 2,000 PSA simulations. In contrast, standard care (two human readers), AI-based double reading (one human plus AI), and triple reading (two humans plus AI) each have a 0% probability of being most cost-effective. This strong performance of the AI-assisted strategy persists across all thresholds up to £100,000, with a 99% probability of being most cost-effective at a £50,000 threshold and an 88% probability at a £100,000 threshold.

As the willingness-to-pay threshold approaches £100,000, the likelihood that double reading by one human plus AI is most cost-effective increases modestly, from 4.2% at £75,000 to 9.4% at £100,000. In contrast, the probability that triple reading (two humans plus AI) is most cost-effective remains below 1% across this range, and standard care is only slightly higher, reaching 2.3% at £100,000. Overall, these results suggest that, across all thresholds considered, any strategy other than a single read with AI is unlikely to be most cost-effective.

Table A32. Probability strategies are most cost-effective by cost per QALY threshold

| Threshold | Standard double reading by two human readers | Double reading by one human plus AI | Triple reading by two humans plus AI. | Single read by AI |
| --- | --- | --- | --- | --- |
| 0 | 0.0% | 0.0% | 0.0% | 100.0% |
| £10,000 | 0.0% | 0.0% | 0.0% | 100.0% |
| £20,000 | 0.0% | 0.0% | 0.0% | 100.0% |
| £25,000 | 0.0% | 0.0% | 0.0% | 100.0% |
| £30,000 | 0.0% | 0.1% | 0.0% | 99.9% |
| £35,000 | 0.0% | 0.2% | 0.0% | 99.9% |
| £40,000 | 0.0% | 0.3% | 0.0% | 99.7% |
| £45,000 | 0.0% | 0.5% | 0.1% | 99.5% |
| £50,000 | 0.0% | 0.7% | 0.1% | 99.3% |
| £75,000 | 0.7% | 4.2% | 0.2% | 95.1% |
| £100,000 | 2.3% | 9.4% | 0.4% | 88.0% |

The Net Monetary Benefit (NMB) for each screening strategy across a range of cost-effectiveness thresholds is reported in Table A33. Overall, the results indicate that a single reading with is the most consistently most cost-effective strategy across typical WTP thresholds, with a clear advantage over standard care and triple reading, particularly at the thresholds used in the UK (£20,000 to £30,000). The next most cost effective option is double reading with one human plus AI.

Table A33. Net Monetary Benefit by threshold and screening strategy

| Threshold | NMB for standard double reading by two human readers | NMB for double reading by one human plus AI | NMB for triple reading by two humans plus AI. | NMB for single read by AI. |
| --- | --- | --- | --- | --- |
| 0 | - £7,668 | - £7,637 | - £7,741 | - £7,508 |
| £20,000 | £284,158 | £284,193 | £284,090 | £284,319 |
| £25,000 | £357,114 | £357,150 | £357,047 | £357,276 |
| £30,000 | £430,071 | £430,107 | £430,005 | £430,233 |
| £35,000 | £503,027 | £503,065 | £502,962 | £503,190 |
| £45,000 | £648,940 | £648,979 | £648,877 | £649,103 |
| £55,000 | £794,853 | £794,894 | £794,793 | £795,017 |
| £65,000 | £940,766 | £940,809 | £940,708 | £940,931 |
| £75,000 | £1,086,678 | £1,086,724 | £1,086,623 | £1,086,844 |
| £100,000 | £1,451,461 | £1,451,510 | £1,451,411 | £1,451,629 |

A single read by AI strategy is found to be highly cost-effective compared to all strategies. In the following appendix subsection, we provide the comparative effectiveness when this strategy is excluded for consideration in the analysis, in order to further detail the effectiveness of the other two strategies when compared to standard care.

**Scenario Analysis. Probabilistic Results with Single reading by AI strategy excluded.**

It is possible that the policy considers the Single Reading by AI strategy impractical. Therefore, in Table A34 and Figure A4, we present below a scenario analysis showing the probabilistic results without this intervention, to determine which of the two remaining AI strategies may be more cost-effective.

At very low thresholds (e.g., £0 per QALY), double reading with one human and AI is overwhelmingly favoured, with a 91.45% probability of being most cost-effective, while standard care (two human readers) holds only an 8.55% probability, and triple reading (two humans plus AI) has a 0% probability. This strong performance of the AI-assisted strategy persists across all thresholds up to £100,000, though with gradually declining dominance.

As the WTP threshold increases, the probability that standard care is most cost-effective rises slightly, from 8.55% at £0 to 26.85% at £100,000. In contrast, the probability for the 1 human with AI strategy decreases from 91.45% to 65.65% over the same range, indicating that although it remains the most likely cost-effective option across all thresholds, the margin narrows.

Notably, the triple reading strategy (2 humans + AI) begins with 0% probability of being most cost-effective at thresholds up to £20,000, but its probability increases slowly at higher thresholds, reaching 7.5% at £100,000. This suggests that while it is not generally most cost-effective at standard WTP levels (e.g., £20,000–£30,000), it may become viable in high-resource settings or under specific policy preferences valuing marginal health gains.

Table A34. Probability strategies are most cost-effective by cost per QALY threshold

| Threshold | Standard double reading by two human readers | Double reading by one human plus AI | Triple reading by two humans plus AI. |
| --- | --- | --- | --- |
| 0 | 8.55% | 91.45% | 0.00% |
| £10,000 | 9.90% | 90.10% | 0.00% |
| £20,000 | 11.95% | 88.00% | 0.05% |
| £25,000 | 13.75% | 86.20% | 0.05% |
| £30,000 | 15.25% | 84.70% | 0.05% |
| £35,000 | 16.55% | 83.30% | 0.15% |
| £40,000 | 17.75% | 81.90% | 0.35% |
| £45,000 | 19.40% | 80.20% | 0.40% |
| £50,000 | 20.85% | 78.50% | 0.65% |
| £75,000 | 24.85% | 71.20% | 3.95% |
| £100,000 | 26.85% | 65.65% | 7.50% |

Figure A4. Cost effectivness acceptability curve showing the probability strategies are most cost-effective by cost per QALY threshold, when the Single Reading by AI strategy is excluded.

**Probabilistic sensitivity analysis: number of runs**

In conducting probabilistic sensitivity analysis for this health economic decision model, the number of PSA runs was decided by examining the estimated net monetary benefit (NMB) variance across the three interventions: Standard Care (standard double reading by two humans), double reading by one human plus AI, triple reading by two humans plus AI and a single reading by AI. As the number of PSA runs increases from 100 to 2000, there is a marked decline in the variance of the mean NMB and its associated standard error, indicating greater stability and reliability in the results. Initially, at lower PSA runs (e.g., 100 runs), the variance and standard errors are high (around £19 million variance and 436 standard error), reflecting substantial uncertainty in the estimates. With increasing simulations, variance steadily decreases, plateauing near 2000 runs, where the variance in mean NMB stabilizes around £15.3 million with a standard error of approximately £87 for all three interventions. This convergence shows that 2000 PSA runs provide a sufficiently precise estimate of the mean NMB variance to support decision-making. The results of this testing are presented in Table A35.

Table A35. Net Monetary Benefit by threshold and screening strategy

| Number of PSA runs | Standard Care (Standard double reading by two human readers) | | Double reading by one human plus AI | | Triple reading by two humans plus AI. | | Single read by AI | |
| --- | --- | --- | --- | --- | --- | --- | --- | --- |
|  | Variance in mean NMB @ £20,000 (£) | Standard error (£) | Variance in mean NMB @ £20,000 | Standard error | Variance in mean NMB @ £20,000 | Standard error | Variance in mean NMB @ £20,000 | Standard error |
| 100 | 19035493 | 436 | 19019097 | 436 | 19025440 | 436 | 19018978 | 436 |
| 200 | 17494015 | 296 | 17483678 | 296 | 17501032 | 296 | 17477965 | 296 |
| 300 | 17170101 | 239 | 17156194 | 239 | 17171540 | 239 | 17151640 | 239 |
| 400 | 16872647 | 205 | 16865242 | 205 | 16878879 | 205 | 16856385 | 205 |
| 500 | 17039993 | 185 | 17036230 | 185 | 17047041 | 185 | 17022786 | 185 |
| 600 | 16189331 | 164 | 16190036 | 164 | 16198143 | 164 | 16174214 | 164 |
| 700 | 16201091 | 152 | 16205081 | 152 | 16213558 | 152 | 16190821 | 152 |
| 800 | 16122107 | 142 | 16128015 | 142 | 16139983 | 142 | 16114324 | 142 |
| 900 | 15958896 | 133 | 15963621 | 133 | 15972065 | 133 | 15950158 | 133 |
| 1000 | 16099041 | 127 | 16100487 | 127 | 16113434 | 127 | 16093397 | 127 |
| 1100 | 16032991 | 121 | 16037353 | 121 | 16047720 | 121 | 16030791 | 121 |
| 1200 | 15907606 | 115 | 15911092 | 115 | 15921845 | 115 | 15904656 | 115 |
| 1300 | 15638067 | 110 | 15643416 | 110 | 15653621 | 110 | 15637048 | 110 |
| 1400 | 15512298 | 105 | 15516764 | 105 | 15525256 | 105 | 15508463 | 105 |
| 1500 | 15400630 | 101 | 15405785 | 101 | 15413747 | 101 | 15397172 | 101 |
| 1600 | 15363276 | 98 | 15367482 | 98 | 15374956 | 98 | 15356927 | 98 |
| 1700 | 15418033 | 95 | 15421898 | 95 | 15428698 | 95 | 15410649 | 95 |
| 1800 | 15315679 | 92 | 15318589 | 92 | 15326368 | 92 | 15307408 | 92 |
| 1900 | 15126737 | 89 | 15130593 | 89 | 15138576 | 89 | 15119664 | 89 |
| 2000 | 15295419 | 87 | 15299597 | 87 | 15308638 | 87 | 15291040 | 87 |

**Probabilistic sensitivity analysis: Distributions and parameters**

In the probabilistic sensitivity analysis, each input parameter was assigned a statistical distribution based on the nature of the data and the available evidence. The assignment of distributions and the specific variables to which they were applied are outlined here.

All variables representing probabilities or proportions bounded between zero and one were sampled from either a Beta or Dirichlet distribution. For individual probabilities, the Beta distribution was applied. Where empirically-informed standard errors (such as those derived from 95% confidence intervals or published standard errors) were available, these were used to parameterise the Beta distribution. This included variables such as the sensitivity and specificity of mammography, stratified by BI-RADS breast density and age group, as well as multipliers reflecting the effect of artificial intelligence on sensitivity and specificity. Health utility or QALY decrements associated with distinct health states, such as the disutility from symptomatic cancer, ductal carcinoma in situ (DCIS), or cancer stages two to four, were also treated this way. In cases where direct measures of uncertainty were not available, an assumed coefficient of variation of 10 percent (meaning the standard error equals 0.1 times the mean) was used. This assumption was applied to annual age-specific mortality rates derived from life-tables, annual probabilities of DCIS and invasive recurrence after initial diagnosis, cancer incidence probabilities by age group, true-positive and false-positive probabilities for biopsy procedures, probabilities of further assessment, biopsies and imaging after initial screening, and per-screen attendance probabilities.

Where probabilities or proportions within a group were required to sum to one, the Dirichlet distribution was used. This approach was taken for the stage-mix distribution of cancer at diagnosis (such as local, regional, or distant stages) by age group and by mode of detection (screen-detected versus symptomatic), as well as for the distribution of age at diagnosis among screen-detected cancers. For the Dirichlet distribution, the concentration parameter alpha was specified as the product of the raw probability and a pseudo-count, as appropriate for modelling proportional outcomes based on assumed sample sizes.

Variables representing continuous, strictly positive values, such as costs and standard deviations, were assigned Gamma distributions assuming a 10 percent standard error. This included all unit costs associated with screening and diagnosis, including mammography, ultrasound, magnetic resonance imaging, general practitioner visits, biopsy procedures, and the costs of sending invitations, were assigned Gamma distributions. Artificial intelligence license fees and integration or governance costs.

A standard Normal distribution, defined by a mean and standard deviation and possibly allowing rare negative values due to statistical uncertainty, was used for the mean breast density among women without cancer, with standard error estimated from data. The mean breast density among women with screen-detected cancer was similarly assigned a Normal distribution. The incremental or “extra” decrement in breast density for women with screen-detected cancer was modelled using a standard deviation set at 10 percent of the absolute value of the mean. The artificial intelligence intervention multipliers for sensitivity and specificity were constrained to be non-negative in a truncated Normal.

After probabilistic sampling of the above, any remaining parameters treated as purely deterministic. These constants included age of eligibility for screening, such as women aged fifty to sixty-nine, and the fixed interval between screening rounds, as determined by policy or guidelines, for example, biennial or triennial screening. The background all-cause mortality rates and cancer incidence rate were not varied in sensitivity analyses. Health utility values for the general population (i.e. non cancer health states) were treated as constants. In cases where direct measures of uncertainty were not available, an assumed coefficient of variation of 10 percent (meaning the standard error equals 0.1 times the mean) was used. Table A36 shows the standard errors of the parameters that had an empirically informed standard error.

Table A36. Standard errors of parameters with empirically informed estimates

| **Description** | **Distribution** | **Mean** | **Standard Error / coefficient of variation** |
| --- | --- | --- | --- |
| Mean breast density among women with screen-detected cancer aged 45–49 | Normal | 13.4916 | 1.0514 |
| Mean breast density among women with screen-detected cancer aged 50–54 | Normal | 10.7216 | 0.71422 |
| Mean breast density among women with screen-detected cancer aged 55–59 | Normal | 7.81139 | 0.40219 |
| Mean breast density among women with screen-detected cancer aged 60–64 | Normal | 7.45076 | 0.33669 |
| Mean breast density among women with screen-detected cancer aged 65–69 | Normal | 6.68333 | 0.24408 |
| Mean breast density among women with screen-detected cancer aged 70–75 | Normal | 7.01291 | 0.32157 |
| Multiplier on mammography sensitivity for double reading by one human plus AI in breast-density band A | Trunc-Normal | 1 | 0.04847 |
| Multiplier on mammography sensitivity for double reading by one human plus AI in breast-density band B | Trunc-Normal | 1 | 0.04847 |
| Multiplier on mammography sensitivity for double reading by one human plus AI in breast-density bands C+D | Trunc-Normal | 1 | 0.04847 |
| Multiplier on mammography sensitivity for triple reading by two humans plus AI in breast-density band A | Trunc-Normal | 1.03333 | 0.09928 |
| Multiplier on mammography sensitivity for triple reading by two humans plus AI in breast-density band B | Trunc-Normal | 1.0678 | 0.11504 |
| Multiplier on mammography sensitivity for triple reading by two humans plus AI in breast-density bands C+D | Trunc-Normal | 1.09804 | 0.10778 |
| Multiplier on mammography false positive rate for double reading by one human plus AI relative to baseline | Trunc-Normal | 0.93909 | 0.00959 |
| Multiplier on mammography false positive rate for triple reading by two humans plus AI relative to baseline | Trunc-Normal | 1.04931 | 0.01049 |

**Appendix References**

NHS Digital. (2024, January 30). Breast Screening Programme, England 2022–23: Table 4 – number of women invited and uptake of invitations to screen, by age band and type of invitation [Statistical publication]. NHS Digital. Retrieved from

Johns LE, Moss SM; Trial Management Group. Randomised controlled trial of mammographic screening from age 40 (‘Age’ trial): patterns of screening attendance. *J Med Screen* 2010; 17: 37–43.

Office for National Statistics. National life tables—life expectancy in the UK: 2021 to 2023. Statistical bulletin. Office for National Statistics; 2025 Mar 18. Available from: https://www.ons.gov.uk/peoplepopulationandcommunity/birthsdeathsandmarriages/lifeexpectancies/bulletins/nationallifetablesunitedkingdom/2021to2023 (accessed 3 Jul 2025).

NHS Digital. Breast Screening Programme, England, 2023–24. Statistical bulletin. NHS Digital; 2025 Feb 18. Available from: https://digital.nhs.uk/data-and-information/publications/statistical/breast-screening-programme/england—2023-24. Accessed 3 Jul 2025.

Bhatt R, van den Hout A, Antoniou AC, Shah M, Ficorella L, Steggall E, et al. Estimation of age of onset and progression of breast cancer by absolute risk dependent on polygenic risk score and other risk factors. *Cancer* 2024; 130: 1590–9.

Isheden G, Humphreys K. Modelling breast cancer tumour growth for a stable disease population. *Stat Methods Med Res* 2019; 28: 681–702.

Chua AV, Sheng H, Liang E, et al. Epidemiology of early vs late recurrence among women with early stage estrogen receptor–positive breast cancer in the Pathways Study. *JNCI J Natl Cancer Inst* 2024; 116: 1621–31.

Morgan E, O’Neill C, Shah R, et al. Metastatic recurrence in women diagnosed with non-metastatic breast cancer: a systematic review and meta-analysis. *Breast Cancer Res* 2024; 26: 171.

Hassett MJ, Uno H, Cronin AM, et al. Survival after recurrence of stage I–III breast, colorectal, or lung cancer. *Cancer Epidemiol* 2017; 49: 186–9

Mannu GS, Wang Z, Broggio J, et al. Invasive breast cancer and breast cancer mortality after ductal carcinoma in situ diagnosed through screening: population based observational cohort study. *BMJ* 2020; 369: m1570.

National Audit of Breast Cancer in Older Patients (NABCOP). 2020 Annual Report: results of the prospective audit in England and Wales for women diagnosed between January 2014 and December 2018. Clinical Effectiveness Unit, Royal College of Surgeons of England; 2020. Available from: https://www.nabcop.org.uk/reports/nabcop-2020-annual-report/. Accessed 3 Jul 2025.

National Audit of Breast Cancer in Older Patients (NABCOP). 2022 Annual Report: results of the prospective clinical audit of care received by women diagnosed with breast cancer in England and Wales between 1 January 2014 and 31 December 2020. Clinical Effectiveness Unit, Royal College of Surgeons of England; 2022 May. Available from: https://www.nabcop.org.uk/reports/nabcop-2022-annual-report/. Accessed 3 Jul 2025

Dembrower K, Crippa A, Colón E, Eklund M, Strand F. Artificial intelligence for breast cancer detection in screening mammography in Sweden: a prospective, population-based, paired-reader, non-inferiority study. *Lancet Digit Health* 2023; 5: e703–11.

Payne NR, Hickman SE, Black R, Priest AN, Hudson S, Gilbert FJ. Breast density effect on the sensitivity of digital screening mammography in a UK cohort. *Eur Radiol* 2025; 35: 177–87.

British Society of Breast Radiology, NHS England, Public Health England, The Royal College of Radiologists. Breast imaging and diagnostic workforce in the United Kingdom: results of a survey of NHS Breast Screening Programme units and radiology departments (Ref. No. BFCR(16)2). London: The Royal College of Radiologists; 2016. Available from: https://www.rcr.ac.uk/our-services/all-our-publications/clinical-radiology-publications/the-breast-imaging-and-diagnostic-workforce-in-the-united-kingdom. Accessed 23 Jun 2025.

NHS Employers. Pay and Conditions Circular (M&D) 4/2023: pay award for hospital medical and dental staff, doctors and dentists in public health, the community health service and salaried primary dental care (England). NHS Employers; 2023 Aug 7. Available from: https://www.nhsemployers.org/system/files/2023-08/Pay%20and%20Conditions%20Circular%20%28MD%29%204-2023%20FINAL_0.pdf. Accessed 20 Jun 2025.

The Royal College of Radiologists. Clinical Radiology Census Report 2023. The Royal College of Radiologists; 2023. Available from: https://www.rcr.ac.uk/news-policy/policy-reports-initiatives/clinical-radiology-census-reports/. Accessed 20 Jun 2025.

Society of Radiographers. Diagnostic Radiography Workforce UK Census 2021. Society of Radiographers; 2021. Available from: https://www.sor.org/download-file?f=9dbc9e49-4b03-4e6c-83cf-bade28aef1bb&t=a. Accessed 3 Jul 2025.

British Medical Association. Pay and Conditions Circular MD-4-2023. British Medical Association; 2023. Available from: https://www.bma.org.uk/media/sfngdzmc/pay-and-conditions-circular-md-4-2023-final_0.pdf. Accessed 3 Jul 2025.

Taylor-Phillips S, Wallis MG, Jenkinson D, et al. Effect of using the same vs different order for second readings of screening mammograms on rates of breast cancer detection: a randomized clinical trial. *JAMA* 2016; 315: 1956–65.

National Institute for Health and Care Excellence (NICE). Artificial intelligence in mammography. Medtech innovation briefing [MIB242]. NICE; 2021 Jan 5. Available from: https://www.nice.org.uk/advice/mib242/resources/artificial-intelligence-in-mammography-pdf-2285965629587653. Accessed 3 Jul 2025.

Personal Social Services Research Unit (PSSRU). Unit Costs of Health and Social Care 2023 Manual. Technical report. University of Kent & University of York; 2023. Available from: https://kar.kent.ac.uk/105685. Accessed 3 Jul 2025.

East Midlands Radiology Consortium (EMRAD). AI in Breast Screening Evaluation: full technical report. NHS EMRAD; 2020. Available from: https://emrad.nhs.uk/images/AI_in_Breast_Screening_Evaluation_Final_Report_-_Full_technical.pdf. Accessed 21 Jun 2025.

Wills L, Nagarwalla D, Pearson C, et al. Estimating surgery, radiotherapy and systemic anti-cancer therapy treatment costs for cancer patients by stage at diagnosis. *Eur J Health Econ* 2024; 25: 763–74.

Wilkinson AN, Seely JM, Rushton M, et al. Capturing the true cost of breast cancer treatment: molecular subtype and stage-specific per-case activity-based costing. *Curr Oncol* 2023; 30: 7860–73.

Laudicella M, Walsh B, Burns E, Smith PC. Cost of care for cancer patients in England: evidence from population-based patient-level data. *Br J Cancer* 2016; 114: 1286–92.

Diernberger K, Luta X, Bowden J, et al. Variation in hospital cost trajectories at the end of life by age, multimorbidity and cancer type. *Int J Popul Data Sci* 2023; 8: 1.

Gidlow CJ, Ellis NJ, Riley V, et al. Randomised controlled trial comparing uptake of NHS Health Check in response to standard letters, risk-personalised letters and telephone invitations. *BMC Public Health* 2019; 19: 1–11.

Vlahiotis A, Griffin B, Stavros AT, et al. Analysis of utilization patterns and associated costs of the breast imaging and diagnostic procedures after screening mammography. *Clinicoecon Outcomes Res* 2018; 10: 157–67.

NHS England. 2022–23 National Cost Collection Data Publication. NHS England; 2024 Jul 9. Available from: https://www.england.nhs.uk/costing-in-the-nhs/national-cost-collection/. Accessed 3 Jul 2025.

Shen Y, Winget M, Yuan Y, et al. The impact of false positive breast cancer screening mammograms on screening retention: a retrospective population cohort study in Alberta, Canada. *Can J Public Health* 2017; 108: e539–45.

McNamara S, Schneider PP, Love-Koh J, et al. Quality-adjusted life expectancy norms for the English population. *Value Health* 2023; 26: 163–69.

Bromley HL, Mann GB, Petrie D, et al. Valuing preferences for treating screen detected ductal carcinoma in situ. *Eur J Cancer* 2019; 123: 130–37.

Sun L, Cromwell D, Dodwell D, et al. Costs of early invasive breast cancer in England using national patient-level data. *Value Health* 2020; 23: 1042–50.

Wang Y, Gavan SP, Steinke D, et al. The impact of age on health utility values for older women with early-stage breast cancer: a systematic review and meta-regression. *Health Qual Life Outcomes* 2022; 20: 169.

Matza S, Howell TA. Fung ET, Janes SM, Seiden M., Hackshaw A., Nadauld L, Karn H. and Chung, K.C., 2024. Health State Utilities Associated with False-Positive Cancer Screening Results. PharmacoEconomics-Open, 8(2), pp.263-276.

Moshina N, Falk RS, Botteri E, et al. Quality of life among women with symptomatic, screen-detected, and interval breast cancer, and for women without breast cancer: a retrospective cross-sectional study from Norway. *Qual Life Res* 2022; 31: 1057–68.

Roine E, Sintonen H, Kellokumpu-Lehtinen PL, et al. Long-term health-related quality of life of breast cancer survivors remains impaired compared to the age-matched general population especially in young women: results from the prospective controlled BREX exercise study. *Breast* 2021; 59: 110–16.

Rautalin M, Färkkilä N, Sintonen H, et al. Health-related quality of life in different states of breast cancer—comparing different instruments. *Acta Oncol* 2018; 57: 622–28.

Vlahiotis A, Griffin B, Stavros AT, et al. Analysis of utilization patterns and associated costs of the breast imaging and diagnostic procedures after screening mammography. *Clinicoecon Outcomes Res* 2018; 10: 157–67.

Shen Y, Winget M, Yuan Y, et al. The impact of false positive breast cancer screening mammograms on screening retention: a retrospective population cohort study in Alberta, Canada. *Can J Public Health* 2017; 108: e539–45.

NHS Digital. Cancer Survival in England: cancers diagnosed 2016 to 2020, followed up to 2021. National statistics. NHS Digital; 2023 Feb 16. Available from: https://digital.nhs.uk/data-and-information/publications/statistical/cancer-survival-in-england/cancers-diagnosed-2016-to-2020-followed-up-to-2021. Accessed 3 Jul 2025.

Narod SA, Iqbal J, Giannakeas V, et al. Breast cancer mortality after a diagnosis of ductal carcinoma in situ. *JAMA Oncol* 2015; 1: 888–96.

Ali AMG, Greenberg D, Wishart GC, Pharoah P. Patient and tumour characteristics, management, and age-specific survival in women with breast cancer in the East of England. *Br J Cancer* 2011; 104: 564–70.

Buschmann L, Wellmann I, Bonberg N, et al. Isolating the effect of confounding from the observed survival benefit of screening participants—a methodological approach illustrated by data from the German mammography screening programme. *BMC Med* 2024; 22: 43.

Shen Y, Yang Y, Inoue LY, et al. Role of detection method in predicting breast cancer survival: analysis of randomized screening trials. *JNCI J Natl Cancer Inst* 2005; 97: 1195–203.

Damiani C, Kalliatakis G, Sreenivas M, et al. Evaluation of an AI model to assess future breast cancer risk. *Radiology* 2023; 307: e222679
